# Wastewater-based epidemiology: deriving a SARS-CoV-2 data validation method to assess data quality and to improve trend recognition

**DOI:** 10.3389/fpubh.2024.1497100

**Published:** 2024-12-12

**Authors:** Cristina J. Saravia, Peter Pütz, Christian Wurzbacher, Anna Uchaikina, Jörg E. Drewes, Ulrike Braun, Claus Gerhard Bannick, Nathan Obermaier

**Affiliations:** ^1^Wastewater Technology Research, Wastewater Disposal, German Environment Agency, Berlin, Germany; ^2^Infectious Disease Epidemiology, Surveillance, Robert-Koch-Institute, Berlin, Germany; ^3^Chair of Urban Water Systems Engineering, Technical University of Munich, Garching, Germany; ^4^Wastewater Analysis, Monitoring Methods, German Environment Agency, Berlin, Germany

**Keywords:** SARS-CoV-2, data plausibility, automated quality control, wastewater-based epidemiology, wastewater treatment plant classification, outlier detection

## Abstract

**Introduction:**

Accurate and consistent data play a critical role in enabling health officials to make informed decisions regarding emerging trends in SARS-CoV-2 infections. Alongside traditional indicators such as the 7-day-incidence rate, wastewater-based epidemiology can provide valuable insights into SARS-CoV-2 concentration changes. However, the wastewater compositions and wastewater systems are rather complex. Multiple effects such as precipitation events or industrial discharges might affect the quantification of SARS-CoV-2 concentrations. Hence, analysing data from more than 150 wastewater treatment plants (WWTP) in Germany necessitates an automated and reliable method to evaluate data validity, identify potential extreme events, and, if possible, improve overall data quality.

**Methods:**

We developed a method that first categorises the data quality of WWTPs and corresponding laboratories based on the number of outliers in the reproduction rate as well as the number of implausible inflection points within the SARS-CoV-2 time series. Subsequently, we scrutinised statistical outliers in several standard quality control parameters (QCP) that are routinely collected during the analysis process such as the flow rate, the electrical conductivity, or surrogate viruses like the pepper mild mottle virus. Furthermore, we investigated outliers in the ratio of the analysed gene segments that might indicate laboratory errors. To evaluate the success of our method, we measure the degree of accordance between identified QCP outliers and outliers in the SARS-CoV-2 concentration curves.

**Results and discussion:**

Our analysis reveals that the flow and gene segment ratios are typically best at identifying outliers in the SARS-CoV-2 concentration curve albeit variations across WWTPs and laboratories. The exclusion of datapoints based on QCP plausibility checks predominantly improves data quality. Our derived data quality categories are in good accordance with visual assessments.

**Conclusion:**

Good data quality is crucial for trend recognition, both on the WWTP level and when aggregating data from several WWTPs to regional or national trends. Our model can help to improve data quality in the context of health-related monitoring and can be optimised for each individual WWTP to account for the large diversity among WWTPs.

## 1 Introduction

The Covid-19 pandemic has underscored the importance of health surveillance of large populations. Having accurate and dependable data on SARS-CoV-2 infection rates is essential for health authorities to implement or adjust measures. Previous projects such as the EU-funded ESI-CorA (Emergency Support Instrument–Detection of SARS-CoV-2 in Wastewater) project have demonstrated that wastewater-based epidemiology (WBE) can be a valuable and cost-efficient addition to clinical surveillance [e.g., (1–7)]. In contrast to clinical surveillance, WBE is independent of the current testing and reporting regime and able to monitor large populations without their active participation. One of the first SARS-CoV-2 wastewater surveillance approaches for few German wastewater treatment plants (WWTP) was initiated as early as November 2020 (8, 9). Through early integration of WBE results into local pandemic management systems, participating health authorities gained the advantage of drawing information from two independent monitoring systems, the clinical surveillance and WBE. The swift transmission of robust results to health authorities is challenging but pivotal. The consolidation, evaluation, and transmission of data from various stakeholders requires significant personnel and time. Consequently, dashboards and corresponding interfaces were developed to facilitate the automated transmission and visualization of data (10). While such automated processes save time and streamline routine tasks, they necessitate integrated quality assurance measures.

At present, the project AMELAG (‘Abwasserbasiertes Monitoring zur Epidemiologischen Lagebewertung’) aims to improve the ongoing SARS-CoV-2 surveillance in Germany and to expand surveillance to other pathogens such as influenza or antimicrobial resistance genomes of bacteria (11). The surveillance infrastructure and data serve as an additional resource for national and regional health authorities to both monitor the spread of pathogens and to offer a permanent platform for the surveillance of future outbreaks. The project supports the development of WWTP specific setups based on common sampling and analysis guidelines but without specifying every aspect of the analysis chain such as the analysis kits used by laboratories. Within the project, more than 150 WWTPs collect samples biweekly at the WWTPs’ inflows. The samples are then analysed in 22 laboratories for SARS-CoV-2 with various methods that are based on Polymerase Chain Reaction (PCR). WWTPs and laboratories provide additional parameter for the samples, such as the daily inflow to the plant (Q) or the electrical conductivity (EC). The participating WWTPs differ from each other in many aspects such as capacity, the sewer system expansion, combined or separated sewer systems, and population density.

Several studies with different sampling and analytical concepts have proven that WBE can be mapped onto other SARS-CoV-2 proxies such as the 7-day-incidence rate (1, 12–15). Trends of both proxies are similar. Most of these studies compare mid-term to long-term data but few of them focus on the predictive capabilities of WBE. However, these predictive capabilities are key when WBE should act as an early warning system (16). Many studies focus on quality control along the steps of sampling and analysis and aim to establish standardised protocols [e.g., (1, 17)].

The 7-day-incidence rate serves as a relatively precise indicator for WBE model fits, particularly during the initial stages of the pandemic when testing and reporting was mandatory. However, this precision diminished as the pandemic progressed, vaccination rates increased, and testing and reporting declined subsequently. In consequence, it can be expected that the number of infected persons covered by the 7-day incidence declined and the relationship between the actual concentration of the SARS-CoV-2 gene segments (hereinafter ‘SARS concentration’) in wastewater and the clinical data became more complex to discern. This also means that WBE, and in particular models that attempt to improve the quality of WBE can hardly be verified using the current 7-day-incidence rate or other, previously used proxies. Using historic 7-day-incidence rate data sets is only a suitable option if WWTPs started monitoring early on and include phases of high and low incidence.

However, the model’s success can be evaluated by analysing the SARS concentration curve itself. The development of the SARS concentration in wastewater is assumed to be smooth and not subject to abrupt changes, hence we expect steady curves. Nevertheless, a “steady” curve is subjective and a quantifiable parameter is needed to evaluate the “steadiness.” The reproduction rate describes the average number of new infections based on a single infection in clinical data. Transferred to WBE, the reproduction rate describes the development of the SARS concentration in wastewater. Huisman et al. (18) found a good accordance between the reproduction rate from wastewater (hereinafter R_w_) and the reproduction rate from clinical cases. In contrast to indicators based on additional sample information, R_w_ is directly derived from SARS concentration data, making it a more straightforward indicator for assessing the plausibility of changes (10). In consequence, we used R_w_ to assess the validity of SARS concentration data points.

The SARS concentrations show short-term fluctuations, their degree varies across different WWTPs. The stronger the fluctuation, the harder it is to identify clear trends. Trends provide a proxy for an increase or decrease of the SARS-CoV-2 prevalence in the population. Significant fluctuations in SARS concentrations may indeed be genuine, influenced by changes in the wastewater composition induced by precipitation or industrial discharges. But they may also stem from errors during sampling, sample preparation, PCR detection, or data transfer. Domestic wastewater is expected to be the primary source of the SARS concentration compared to other types of wastewater such as industrial wastewater or stormwater. In theory, the share and composition of domestic wastewater is fluctuating and most studies recognise the need to process SARS concentration data to account for these fluctuations [e.g., (19–21)]. A common approach is the use of additional sample parameters, which might give information on the share of domestic wastewater, are suitable to identify exceptional events or give a hint on sampling or analysis errors. They can either stem from the WWTP (e.g., Q, pH) or the laboratory (e.g., biomarkers). Hereinafter, we call these additional parameters ‘quality control parameter’ (QCP).

There are basically two possible approaches: the first is to exclude data points that fail a plausibility check due to unusually high or low values in certain QCPs. The second approach is to normalise SARS concentrations with a factor based on, e.g., QCP variation. Normalisation therefore assumes a correlation between the share of domestic wastewater and a parameter and adjusts the SARS concentration. In contrast to that, plausibility checks use exceptional values of parameters to identify and possibly exclude implausible data. Developing a normalisation method proves to be significantly more challenging than evaluating the impacts of QCP plausibility checks. This is largely due to additional uncertainties such as the relation between a QCP and the share of domestic wastewater.

Several studies use different QCPs to approximate the share of domestic wastewater and to calculate the dilution by other types of wastewater. The most common are the daily inflow to the WWTP (Q) (22, 23), wastewater quality parameters (e.g., ammonia, electrical conductivity) (22, 23), biomarkers such as pepper mild mottle virus (PMMoV) (16, 23–25) or population dynamics (23, 26). Specific chemical compounds such as pharmaceuticals or the respective transformation products might have a good approximation of domestic wastewater due to their constant use (27). However, specific chemical QCPs are not part of the routine SARS-CoV-2 monitoring on WWTP and would require additional effort.

The flow to a WWTP typically exhibits only minor fluctuations during dry weather, however, during wet weather events, the flow can change significantly. Therefore, normalisation with the flow might improve the data quality, although it remains unclear if the relation between flow variation and the share of domestic wastewater is linear. Overall, most studies indicate a limited success of normalisation, with results varying widely among WWTPs. For example, Nagarkar et al. (28) show that a normalisation with the flow improved the correlation with clinical data for some WWTPs but it deteriorated for others. The same can be observed for other QCPs. Joseph-Duran et al. (29) state that, e.g., a normalisation with PMMoV does not improve the data whereas Wartell et al. (25) claim that it does. Hence, the implementation of a normalisation method or data plausibility checks requires a comprehensive and WWTP-specific analysis, given the absence of overarching logical relations between parameters.

In addition to wastewater specific QCPs, there are laboratory specific QCPs. The correlation between gene segments (10, 12) serves as a suitable and readily obtainable QCP for validating data points and assessing the precision of PCR-measurements.

Typically, averages (arithmetic or geometric mean) of two or more different SARS-CoV-2 gene segments are used in WBE studies. According to Marques dos Santos et al. (30), a multigene analysis involving three gene segments demonstrates the best fit and the highest correlation with reported active cases. Their analysis suggests that a dual gene approach did not yield significant improvements compared to a single gene analysis. However, these findings are again specific for a WWTP and may not be applicable to other locations. In addition, a gene segment ratio unequal to 1 (e.g., 0.5) between the measured gene segments always creates a weighted mean. Consequently, the means of measurements from different gene combinations cannot be compared to other measurements without using a factor.

However, quantifying multiple gene segments can serve as a quality control for sample analysis, e.g., to detect mutations (10). Furthermore, not calculating a mean adds another level of complexity. With 6 gene segments, there is a theoretical maximum of 63 possible combinations (e.g., N1; N1 and N2; N1, N2, and E, etc.), while 4 gene segments yield 15 possible combinations. Assessing several QCP based on these combinations would result in a large matrix, beneficial for the detailed assessment of a single WWTP but overly complex for multiple WWTPs. Therefore, we calculated the geometric mean of the different gene segments analysed in each sample.

After reviewing the literature, it seems improbable that for different WWTPs, a single parameter is suitable to check the plausibility of SARS concentration data or is able to improve trend recognition through normalisation. Several studies have shown that a thorough analysis of the corresponding sewer system, population dynamics, industrial dischargers etc. of an individual WWTP provides additional information for plausibility checks or helps to choose a parameter for normalisation. However, these analyses are time- and cost-intensive for large datasets such as the AMELAG dataset. Thus, we chose to develop a method that automatically assesses and validates the generated data and is independent of background information for the individual WWTP. Our model aims to identify SARS concentration data that appear implausible and exclude them from the trend calculation. Hence, in a first step, it is imperative to establish criteria which enable an automated evaluation of data plausibility. In a second step, we identified outliers in available quality control parameters (QCP) and compared their coincidence with implausible points in the SARS concentration curves. Then, we selected the QCP with the highest potential to improve the SARS concentration trend recognition based on statistical values. For each WWTP and laboratory, we subsequently used the identified QCP to exclude the corresponding outliers and assessed the change in the SARS concentration trend.

## 2 Materials and methods

This study is premised on the hypothesis that incorporating additional QCPs from both WWTPs and laboratories can enhance the quality of SARS concentration trends in wastewater and bolster the predictive capabilities of WBE. Initially, we elucidate the common sampling and detection methodologies of the research projects analysing WWTP samples and detecting SARS concentrations. We then give a quick overview of the dataset and describe the statistical method that we have implemented in R (31), a software, to identify outliers (see Supplementary material for link to R codes).

### 2.1 Sampling, sample preparation, and detection

In both projects, ESI-CorA and AMELAG, technical guidelines were provided to WWTPs and laboratories, outlining general instructions for sampling, logistics, analysis, and data transfer. In general, 24 h-composite samples were taken either time-or flow-proportionally at the inflow of the WWTPs. A 1 L sample was sent to the laboratory, maintaining it cool. After sample preparation and RNA extraction, SARS detection was carried out with qPCR or dPCR.

Due to the need for a rapid response and the high number of participating WWTPs and laboratories, there is a large variety of applied and accepted methods, e.g., different PCR systems, extraction kits and so on. Detailed information on sampling, sample preparation, and detection is provided in the Supplementary material.

### 2.2 Description of the available data

Alongside the AMELAG dataset, our dataset comprises data obtained from the ESI-CorA research project, the Bay-VOC project (Molecular Genetic SARS-CoV-2 Surveillance Network in the State of Bavaria) as well as the ÖGD-Monitor Rheinland-Pfalz. The data from all projects were then consolidated in a unified database (see Supplementary material for link to input data). In February 2022, ESI-CorA started with 20 WWTPs and the number of WWTPs increased to 158 by January 2024. On average, WWTPs participated for approximately 347 days (SD = 256), with a mean number of 78 samples collected per WWTP. The range of samples taken varied widely, from a minimum of 2 to a maximum of 244 samples, with a standard deviation of 64 samples.

The WWTP added information on sample specific parameters such as Q, the electrical conductivity (EC) and pH-value while laboratories provided data on SARS concentrations and the so called ‘surrogate viruses’ pepper mild mottle virus (PMMoV) and CrAssphage (CrA). Ensuring a minimum level of data quality is essential for evaluating the data from various WWTP and conducting statistical analyses. Overall data quality varies among WWTP due to differences in, e.g., monitoring periods or wastewater composition. The used detection methods vary highly between laboratories; hence WWTP datasets were assessed separately if the analysing laboratory changed during the analysis period. All subsequent analyses are thus WWTP and laboratory (WWTP-Lab) specific.

We initiated our data analysis process by excluding entries outside the analysis period, which ranged from January 1, 2022, to January 31, 2024. Due to the lack of standardisation for limits of detection and quantification, we removed data points if one of the measured gene segments was below the indicated limit of quantification (LOQ). If laboratories did not provide a limit of quantification, we set the limit of quantification to 4,000 gc/L (gene copies per litre), representing the highest LOQ in our associated laboratory. Out of the initial 12,796 data points, 532 were below the LOQ. We excluded all WWTP-Labs with less than 15 SARS concentration measurements (above the LOQ). From the remaining 157 WWTP-Lab datasets, the flow (Q) and the pH-value are the most commonly measured parameters (157 and 140 WWTP-Lab), followed by EC and PMMoV (130 and 122). CrAssphage is reported by significantly fewer WWTP-Labs (28). The most common SARS gene segment is from the envelope protein (E), followed by the nucleocapsid proteins N2 and N1 (106, 102 and 100 WWTP-Labs respectively). The SARS gene segments open reading frame (ORF) and the RNA dependent RNA polymerase (RdRp) are measured in fewer WWTPs (51 and 63); the spike protein (S) gene segment was measured in only 2 WWTPs.

### 2.3 Statistical methods to identify outliers

We identified statistical outliers using an inter quartile range (IQR)-method. Outliers are defined to be either smaller than the lower bound or larger than the upper bound. The calculation of both bounds is as follows:


(1) Lowerbound=Q1−1.5IQR

(2) Upperbound=Q3+1.5IQR

Q1 represents the 25th percentile, IQR the interquartile range (Q3-Q1) and Q3 the 75th percentile. The scale is set to 1.5, which equals a standard deviation of 2.7 from the mean in case of a normal distribution.

Outliers in ratios, such as those of measured gene segments or the reproduction rate must be identified distinctively. Regarding ratios, particularly those being close to 1 or falling below, the standard IQR method tends to underestimate the lower boundary. To address this, we first normalise the respective data by dividing each value through the data sets median to centre it at around 1. We have chosen the median because it is less sensitive to outliers compared with other statistical measures such as the mean. Second, we define the lower boundary to be 1 divided by the upper boundary:


(3) $$\text{Lower}\;\text{bound}\;for\;\text{factors}=\frac{1}{Q3+1.5\;IQR}$$

(4) Upperboundforfactors=Q3+1.5IQR

This procedure disregards the 25th percentile but results in a symmetric definition of outliers. Consequently, both the lower and upper boundaries represent the same ratio.

## 3 Results

First, we analysed and compared the QCPs from WWTPs and laboratories to identify any consistent patterns that could facilitate a standardised check for data plausibility. Subsequently, we evaluated and categorised the WWTP-Lab specific SARS concentration curves by detecting outliers in the reproduction rates and assessing the number of implausible inflection points. We aggregated data from these different WWTP-Lab categories and investigated the uncertainty and informative value of the resulting curves.

Moreover, we conducted a detailed analysis of the QCPs, determining their ranges and flagging any outliers that may indicate extraordinary events or errors leading to potentially unreliable SARS concentrations. After identifying outliers, we assessed the effectiveness of our methodology in identifying implausible SARS concentration data by calculating the F1-score for each QCP.

Finally, we compared the processed data with the original data and examined how both, the WWTP-Lab specific trends and the predictive capabilities of the aggregated dataset have been affected.

### 3.1 WWTP related data: pH-value, EC, Q, surrogates

**Figure 1:**
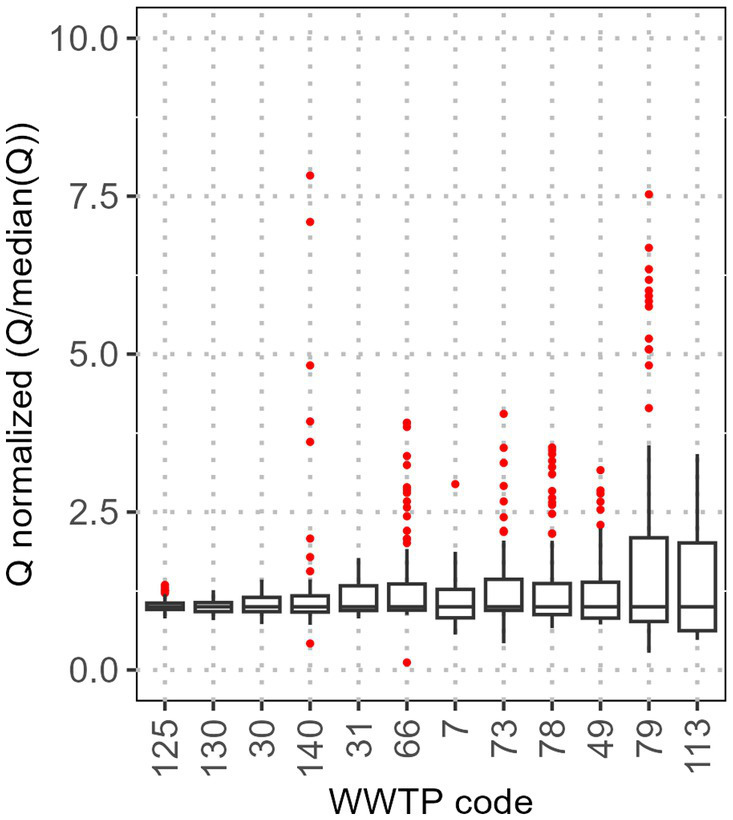
Distribution of the flow at some exemplary WWTP. Q is normalised by the corresponding median and ordered from small to large IQR. Red dots represent statistical outliers.

The participating WWTPs are spread across Germany, exhibiting a wide range of diversity, regarding capacity, geographical and topographical location, sewer systems, connected industry, and commercial dischargers. This diversity is reflected in the quantity, quality, and composition (domestic, industrial, groundwater infiltration, etc.) of the wastewater. The median flow of the participating WWTPs ranges between 16 and 3,545 L/s. As an example, Figure 1 shows the normalised flow (WWTP specific flow divided by its median) for some exemplary WWTPs. While some WWTPs exhibit only minor variations, others show a significantly higher variation, which can be quantified with the IQR of the normalised flow values. The Q1 and Q3 for the IQR of the normalised flow lie at 0.31 and 0.60, respectively.

The QCPs PMMoV, CrAssphage, pH, and EC were normalised with their respective medians and show significant variations between the WWTPs. The data can be found in respective figures in Supplementary Figures S1–S5. For PMMoV, the IQR of the normalised values ranges between 0.82 and 1.27 (Q1 & Q3), for CrAssphage between 0.77 and 1.43. Hence, the so called ‘surrogate’ viruses have larger variations than Q. EC (normalised values: Q1 = 0.19 and Q3 = 0.34) and the pH-value have much smaller variations (normalised values: Q1 = 0.02 and Q3 = 0.05, however, IQR for pH-values are not directly comparable due to the logarithmic scale and are not further considered here).

**Figure 2:**
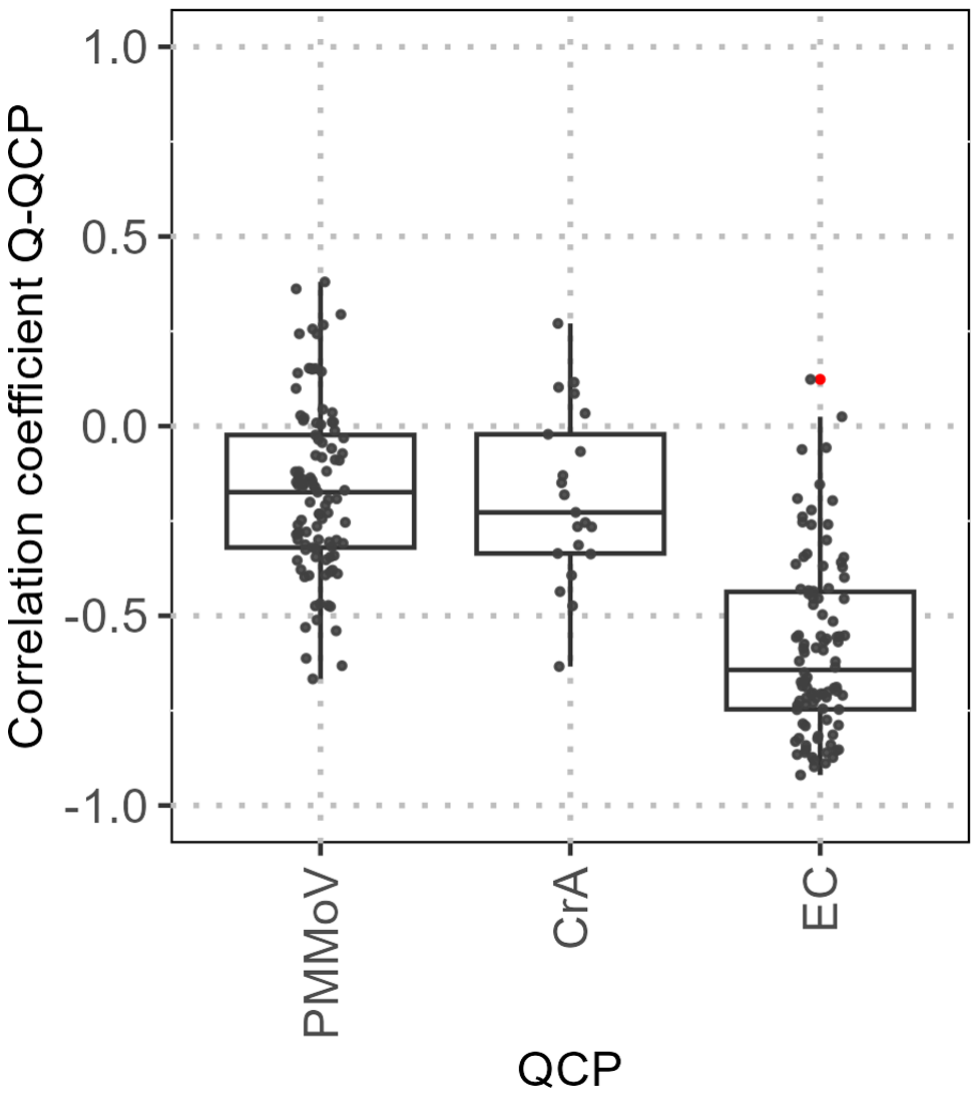
Pearson correlation coefficient for the correlation between Q and QCP for all WWTP. Red dots represent statistical outliers.

We assume that QCPs remain relatively constant in domestic wastewater and change is due to dilution from other wastewater types. Sampling always takes place on the same working days (Monday & Wednesday, usually from the early morning until the early morning of the following day), so we assume a relatively constant amount of domestic wastewater for a given WWTP. Therefore, an increased flow is attributed to non-domestic wastewater and for the QCPs PMMoV, CrAssphage and EC we expect a negative linear correlation with the flow. Figure 2 depicts the Pearson correlation coefficient between the flow and the three QCPs PMMoV, CrAssphage, and EC for each WWTP-Lab where they were analysed. For PMMoV, at most of the WWTP-Labs, the Pearson correlation coefficient is negative with a median of −0.17 (Q1 = −0.32 and Q3 = −0.02). A negative Pearson correlation coefficient is in accordance with the hypothesis that the dilution of domestic wastewater by stormwater or industrial discharges can be observed with the PMMoV concentration. However, most correlation coefficients lie between 0 and −0.5, indicating only a very weak to weak linear relation.

The correlation of CrAssphage with Q is similar to the correlation observed between PMMoV and Q (median: −0.22, Q1 = −0.34 and Q3 −0.02). As anticipated, a negative correlation between these two parameters is evident in a majority (approx. 75%) of the WWTP-Labs. Only very few values lie below −0.5, hence a strong correlation can hardly be seen. For the EC, a good linear correlation (Pearson correlation coefficient < −0.75) to Q can be observed for 23% of the WWTPs. However, there is still a wide range of Pearson correlation coefficients between EC and Q (median −0.64, Q1 = −0.75 and Q3 = −0.44).

### 3.2 Laboratory related data: gene ratios

In contrast to the WWTP related QCPs, an outlier in the gene segment ratio influences the result directly due to the result being the geometric mean of all measured gene segments. The WWTP-Lab specific ratio between the measured gene segments is supposed to be rather constant. Hence, from a theoretical point of view, we expect an almost perfect correlation between different gene segments measured in the same sample.

As with the WWTP related QCPs, there are significant differences between the measured gene segments, the WWTPs, and the laboratories. For example, the Pearson correlation coefficient between two gene segments ranges from a minimum of 0.007 for WWTP 40/laboratory 7/N1 & N2 to a maximum of 0.998 for WWTP 160/laboratory 27/E & N2. Most of the WWTP/laboratory/gene combinations show a high correlation with Pearson correlation coefficients with a median of 0.956 (Q1 = 0.883 and Q3 = 0.986). The Pearson correlation coefficients for all WWTP, laboratory, and gene segment combinations can be found in Supplementary Table S1.

When analysing all correlation data, it becomes evident that no single factor can explain the major differences between the Pearson correlation coefficients. The coefficients vary between different gene segment combinations analysed for the same WWTP and laboratory, they vary for samples from the same WWTP with the same gene segment combination analysed in different laboratories (although they use the same methodical workflow) and for the same laboratory and same gene segment combination analysing samples from different WWTPs.

To further investigate the variability in gene segment ratios, we subsequently examined the 27 laboratories. With some laboratories analysing and reporting more than two gene segments (e.g., E, N1, ORF, RdRp), there are a total of 68 laboratory-specific gene segment combinations (each with 2 gene segments and more than 14 measurements). There are significant differences in both the ratios between two gene segments and the distribution of these ratios. To address this, we normalised the gene segment ratios by dividing them by their mean. While this normalisation is irrelevant at the WWTP level, it facilitates the comparison of different laboratories.

**Figure 3:**
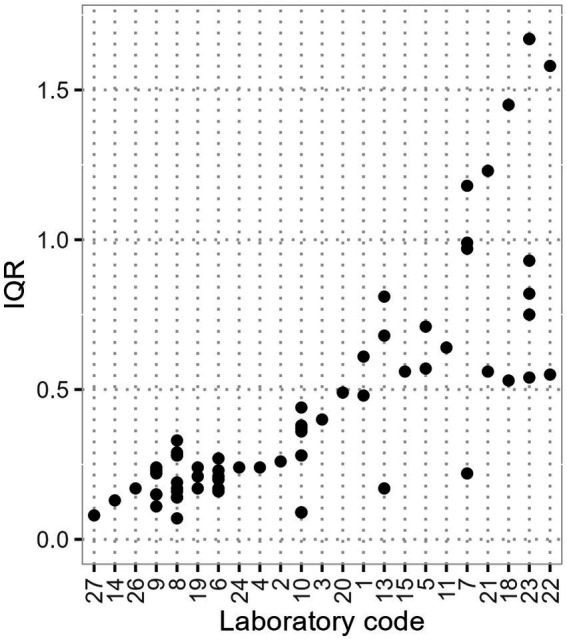
Normalised IQR of the different gene ratios (dots) for the different laboratories. Laboratories 12, 16, 17 and 25 have an IQR of 0 due to measuring a single gene segment or a sum parameter of several gene segments and are not shown here.

Figure 3 displays the IQR of the normalised gene segment ratios (each combination of 2 gene segments is represented as a dot with some laboratories having multiple) of the participating laboratories. A hypothetical IQR value of 0 represents a constant ratio between the gene segments and thus a perfect correlation. A wide IQR suggests poor precision in SARS concentration measurements as fluctuations in the ratio directly influence the calculation of the geometric mean. Consequently, changes in SARS concentration curves may be attributed to laboratory-specific influences. A small normalised IQR suggests that changes in the curve are likely genuine SARS concentration variations and are less likely influenced by laboratory factors.

The IQR of the normalised gene segment ratios has a minimum of 0.07, a 25th percentile of 0.17, a median of 0.28, and a 75th percentile of 0.57 and a maximum of 1.67. Some laboratories, exemplified by laboratory 8, measure different gene segments with varying IQR, ranging from 0.07 for E & N2 to 0.33 for N2 & RdRp. Prioritising gene segments with the highest precision can improve overall data quality.

### 3.3 Trend quality assessment

Besides comparing SARS concentrations in wastewater to clinical data, the WBE trend quality can be evaluated with methods describing the SARS concentration curve. Following the infection dynamics of SARS-CoV-2, we anticipate that the SARS concentration curves exhibit rather smooth transitions without significant fluctuations or abrupt, alternating jumps. The reproduction rate R_w_ is a typical parameter to describe the daily increase/ decrease of SARS-CoV-2 cases in clinical data. According to Huisman et al. (18), the reproduction rate for clinical data is transferable to WBE and does not vary dramatically between different outbreaks. Exceptionally large or small R_w_ represent abrupt jumps in the curve. Based on the assumption that SARS infection dynamics are neither linear nor exponential, we decided to calculate R_w_ for sample date t as the n^th^ root of the quotient of a SARS concentration measurement (SC_t_) and the previous measurement (SC_t-n_), with n being the number of days between both measurements:


(5) $$R_{w,t}=\sqrt[{n}]{\frac{SC_{t}}{SC_{t-n}}}$$

Implausible inflection points (IIP) are data points where a significant increase/decrease of the next measurement follows a significant decrease/increase of the previous data point. A high number of IIPs leads to an unsteady, even ragged SARS concentration curve. We set the boundaries to define IIPs in relation to the distribution of R_w_ for all WWTPs. We defined a SARS concentration as an IIP, if the R_w_ value for the sample date t (R_w,t_) is above the 75th percentile and the subsequent R_w_ value (R_w_ for sample date t + 1, R_w,t + 1_) is below the 25^th^ percentile or vice versa:


(6) $$IIP:R_{w,t}>Q_{R,75\%}\&R_{w,t+1}<Q_{R,25\%}$$

(7) $$IIP:R_{w,t}>Q_{R,25\%}\&R_{w,t+1}<Q_{R,75\%}$$

Unlike assessing a SARS concentration value with R_w_, an implausible inflection must consider the subsequent SARS concentration value. Therefore, it cannot be utilised as a method to assess the validity of the latest SARS concentration value.

R_w_ is calculated for all SARS concentrations in the dataset. Then, the number of R_w_-outliers is calculated with the IQR method described previously. The sum of R_w_-outliers and IIP (R/IIP) are set into relation with the number of total measurements for the WWTP-Labs where SARS concentration measurements representing both an R-outlier and an IIP are only considered once. The share of R/IIP is a proxy for the trend quality of a WWTP-Lab.

To evaluate the impact of the subsequent QCP plausibility check, we analysed the trend quality for each WWTP-Lab individually as described previously. However, in this scenario, we calculated R_w_ using only SARS concentrations of the respective WWTP-Lab and not the whole dataset. This approach provides a higher resolution for the detailed assessment of data quality and the implications of removing data points due to QCP outliers.

We subsequently assessed the trend quality of the WWTP-Labs and divide them into three categories based on the proportion of R/IIPs (each category has 33% of the WWTP-Labs). The results for all WWTP-Labs can be found in Supplementary Table S2.

**Figure 4:**
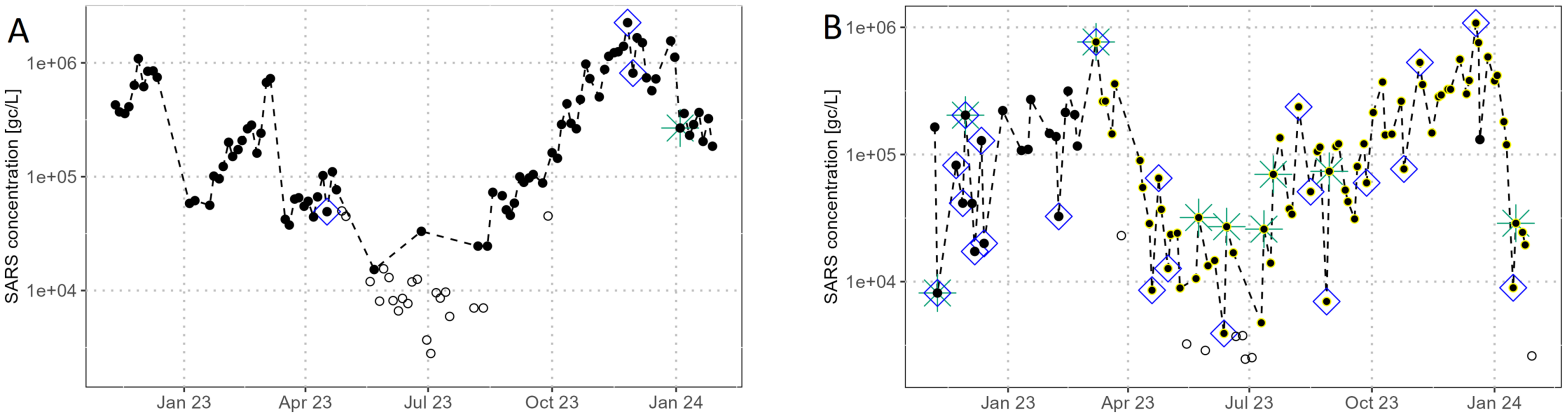
**(A)** SARS concentrations in gene copies per litre at WWTP 139, analysed by laboratory 23, **(B)** SARS concentration in gene copies per litre at WWTP 78, analysed by laboratory 10 (black circles) and laboratory 8 (yellow circles). Logarithmic y-axis. Circles with a white filling represent samples with SARS concentrations below the limit of quantification. Green stars indicate Rw-outliers, blue squares implausible inflection points.

Category 1 represents WWTP-Labs classified as having a ‘good’ trend with outliers ranging from 7 to 19%. Figure 4A displays data from WWTP No.139 and Laboratory No.23 as an example for a trend in category 1. Out of the 85 datapoints, four datapoints are flagged as R/IIPs, three of them representing IIPs. One datapoint is flagged as an R-outlier. The identified R/IIPs are in good accordance with a visual identification of outliers in the SARS concentration curve. The trend appears generally smooth, although some data points in December 2023 exhibit an unsteady curve. Three distinct ‘waves’ can be identified visually, one in December 2022, one in March 2023 and the other from October to December 2023. In summer 2023, most of the measured SARS concentrations lie below the quantification limit and the trend quality cannot be assessed.

Category 2 includes WWTP-Labs with an R/IIP share between 19 and 29%. We classify these as having a ‘mediocre’ quality. Category 3 represents WWTP-Labs we classified as having a ‘bad’ trend with an R/IIP share between 29 and 50%.

Figure 4B shows the trend of WWTP No.78, which was analysed by two laboratories, dividing the trend into two different quality categories. Laboratory No.10 processed samples from February 2022 to February 2023, 31% of the 86 datapoints are R/IIPs and thus exhibit a ‘bad’ data quality (data only shown from December 2022 on). Subsequently, from February 2023 to January 2024, laboratory No.8 analysed samples with 27% of the 71 datapoints classified as R/IIPs. Hence, we classify this trend quality as ‘mediocre’. There are several SARS concentrations below the limit of quantification in the dataset from laboratory No.8. The trend exhibits significant irregularities, characterised by an unsteady curve and multiple isolated spikes comprising concentrations considerably higher or lower than the surrounding values. Many of these spikes have been identified as IIPs, some also as R_w_-outliers. Overall, discerning distinct long-term trends visually is challenging; however, slight ‘waves’ are perceptible in March and autumn 2023.

### 3.4 Effects of data quality on the aggregated SARS concentration curve

**Figure 5:**
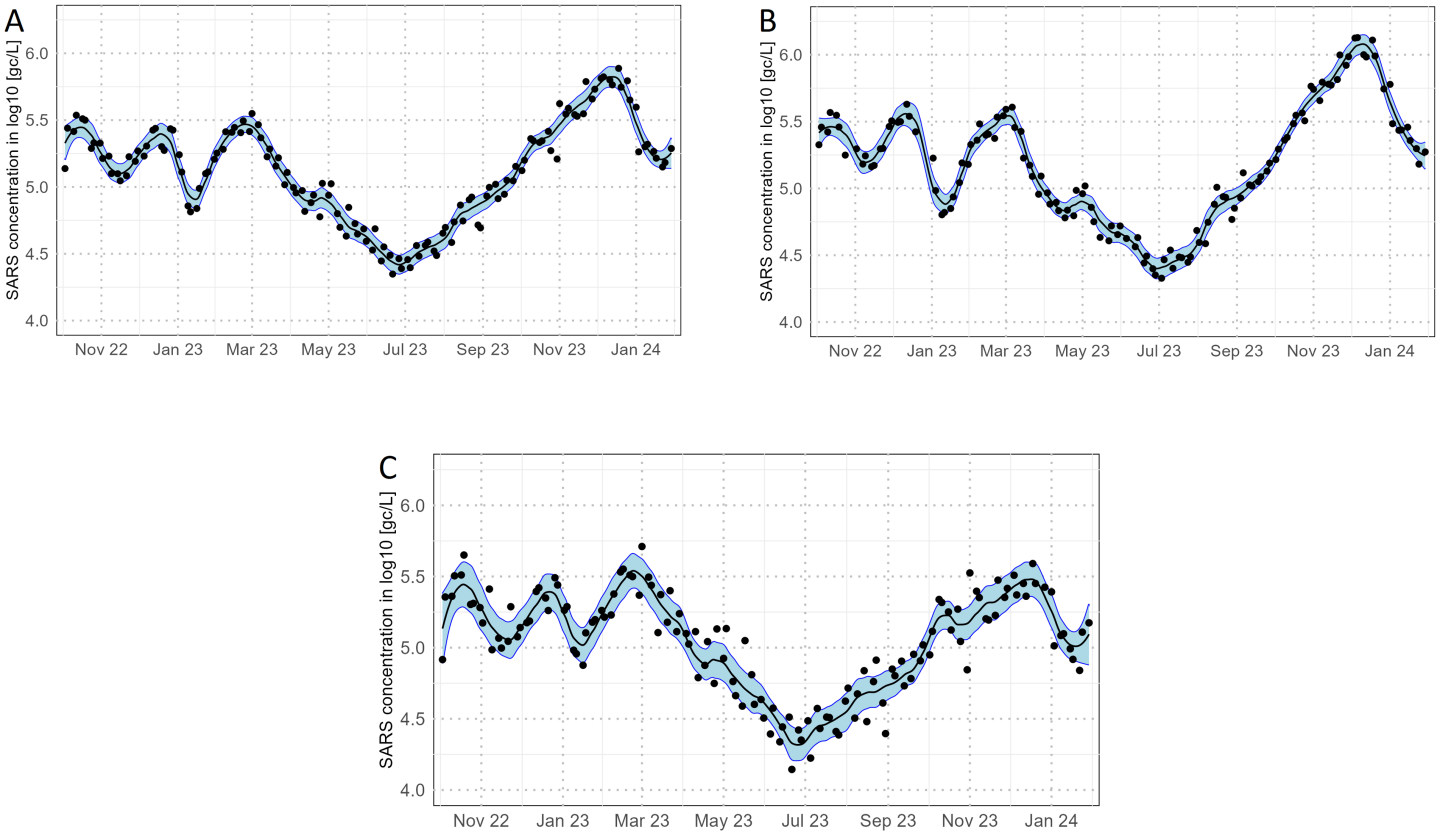
SARS concentration in gene copies per litre aggregated over all sites **(A)**, all sites with ‘good’ data quality **(B)**, and all sites with ‘bad’ data quality **(C)**. Shown are means over WWTP-Lab (dots), a corresponding LOESS regression curve (black line) and associated 95% pointwise confidence intervals (blue area).

We assessed the effects of WWTP-Lab data quality on the precision and informative value of a curve which aggregates information from nearly all WWTP-Labs that participated at the respective time. Therefore, we averaged the log10-transformed SARS concentrations over all WWTP-Labs for each sampling date. As a minimum, each sampling date must have data from at least 10 WWTP-Labs. This procedure reduces the effects of gross outliers that are potentially driven only by a single or few WWTP-Labs. The data points resulting from aggregation, i.e., means, are subsequently smoothed by a LOESS (Locally Estimated Scatterplot Smoothing) regression. Weights are applied to the LOESS regression such that means with higher standard errors receive less weight than means with lower standard errors. The precision of the curve is indicated by confidence bands which are calculated as pointwise 95% confidence intervals (constructed with the corresponding t-distribution quantile) for the smoothed values on the curve. Figure 5A shows the aggregated SARS concentrations when considering all WWTP-Labs. The smooth curve depicts distinct pandemic trends with small deviations of the single mean values from the curve and narrow confidence bands.

A similar picture, albeit with slightly wider confidence bands, arises if only the WWTP-Labs with few R/IIPs, hence good data quality, are considered (Figure 5B). Put differently, the removal of 67% of WWTP-Labs not having a ‘good’ data quality does not have a substantial effect on the informative value of the aggregated curve. However, if only WWTP-Labs with many R/IIPs are considered (‘bad’ data quality), the trend is less distinct, mean values generally deviate more from the curve and the confidence bands become wider (Figure 5C).

### 3.5 Assessing the success of QCP plausibility checks on the WWTP level

**Figure 6:**
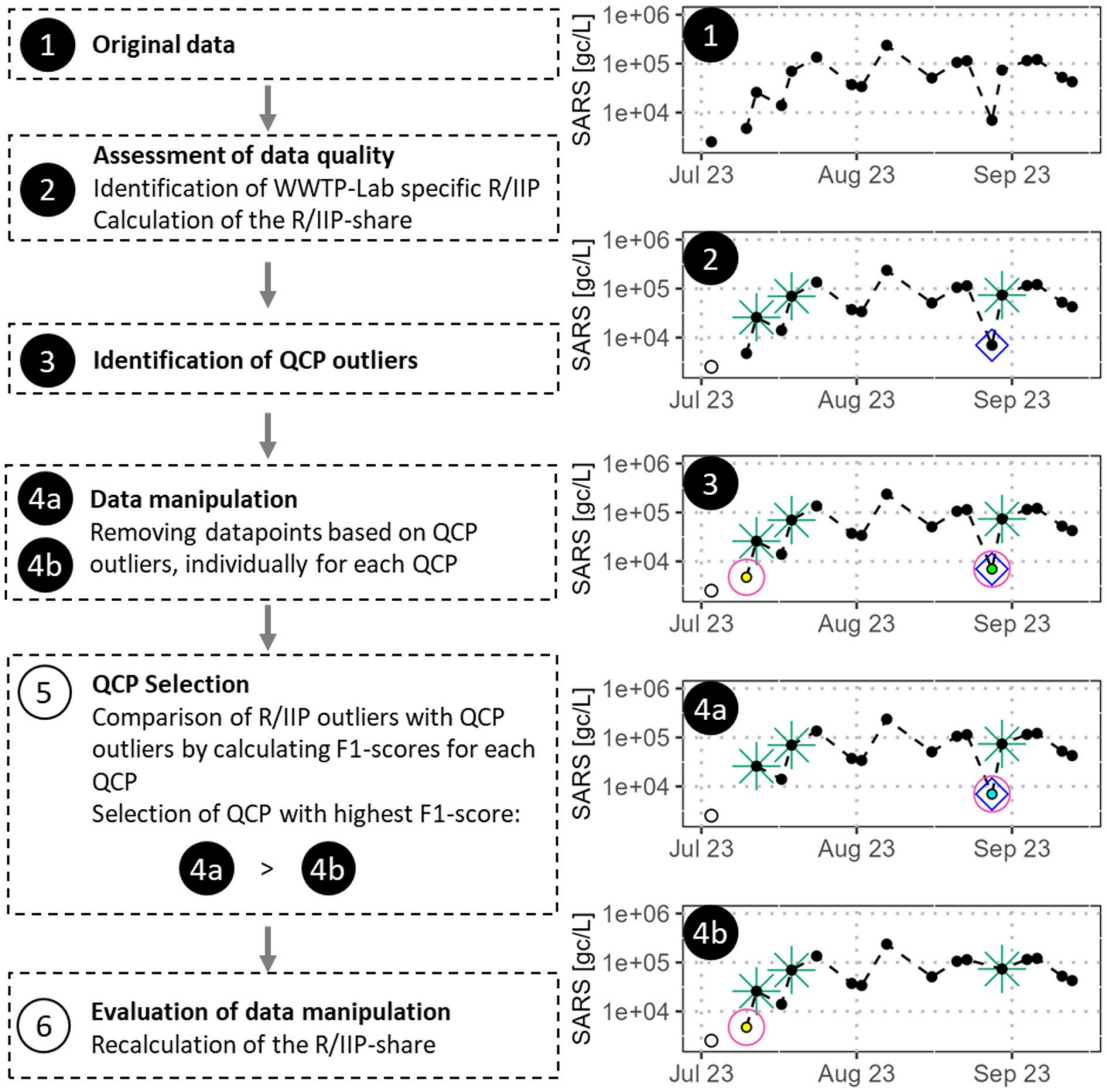
Data processing scheme to identify the QCP with the highest potential to improve the SARS concentration curve. Empty circles show values below the limit of quantification, green stars and blue squares are R/IIP and pink circles highlight outliers in QCP. The different colours indicate the different types of QCP.

The analyses of the aggregated curves in the previous section focused exclusively on R/IIPs and did not consider effects introduced by identifying and removing outliers in QCPs. Figure 6 shows our process to assess the success of these QCP plausibility checks and how to identify the QCP with the highest potential to improve the specific WWTP-Lab data.

Based on the original WWTP-Lab data (step 1), we assessed the data quality (step 2) by identifying R/IIPs (following Equations 5–7, marked as green stars and blue squares) and by calculating their share of all WWTP-Lab datapoints, yielding the original R/IIP share.

Subsequently, we identified potential exceptional events by examining outliers in the QCPs Q, EC, pH, PMMoV, CrA following Equations 1–4 (step 3, highlighted with pink circles, different types of outliers in different colours). The respective QCP may offer insights into the composition of wastewater. We also computed the ratios between the concentrations of all gene segments measured in each sample (gr). Since all gene segments determine the same SARS concentration, their ratios are expected to remain relatively stable. Some laboratories measured up to four different gene segments, resulting in six unique combinations of two gene segments. We classified data points as gene ratio outliers if more than 40% of all gene ratios were outliers (e.g., 2 out of 4, 2 out of 3).

Separately for each QCP, we then removed the identified QCP outliers from the WWTP-Lab dataset (step 4a and step 4b). This leads to several alternative SARS concentrations curves.

For each QCP, we calculated F1-scores (step 5), a statistical indicator, which basically describes how well QCP outliers and R/IIPs coincide. To calculate the F1-score, we assigned the 4 different categories of a confusion matrix based on the definition shown in Table 1.

**Table 1:** Definition of the components of a confusion matrix to assess if our model produces successful results.

True positive (TP)	False positive (FP)
Both, R/IIP and QCP are outliers	No R/IIP outlier, QCP outlier
False negative (FN)	True negative (TN)
R/IIP outlier, no QCP outlier	Both, R/IIP and QCP are no outliers

Based on these categories, we calculated the F1-score as:


$$F1\;\text{score}=\frac{2\;TP}{2\;TP+FP+FN}$$

The F1-score maximises both the chance that potential outliers are identified and that an outlier is an actual outlier. An F1-score of 1 indicates perfect alignment between QCP outliers and R/IIPs, while a score of 0 indicates no alignment between them or that no QCP outliers were identified. For each WWTP, the QCP with the highest F1-score is deemed the most suitable to improve trend quality. Consequently, the best-fitting QCP for a given WWTP-Lab may have a relatively small F1-score, but still outperforms other QCPs in identifying potential R/IIPs.

In a final step (step 6), we assess the data quality change between the original data and the newly generated SARS concentration curve based on the QCP with the highest F1-score identified in step 5. Therefore, we recalculated the R/IIP share of the SARS concentration curve without the QCP outliers and compared it to the original R/IIP share.

**Figure 7:**
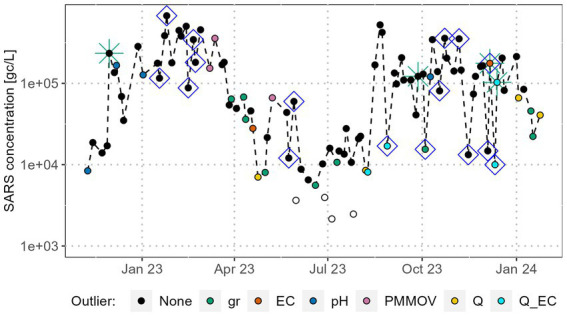
SARS concentration in gene copies per litre at WWTP No.73 with R/IIP and QCP-outliers. Green stars indicate Rw outliers, blue squares IIP. The colour of the datapoints indicates the QCP which was identified as an outlier.

Figure 7 displays the SARS concentration of a selected WWTP from December 2022 to January 2024 with both, R/IIPs and QCP outliers highlighted (outcome of executing step 3). It can be observed that for several datapoints R/IIPs and QCP outliers coincide, especially for prominent R/IIPs in autumn 2023. Nevertheless, there are also many QCP outliers where the SARS concentration aligns well with the curve and R/IIPs which do not reflect in QCP outliers. In the case of this WWTP, the EC achieves the highest F1-score at 0.24, followed by Q (0.16), pH-value (0.06), and the gene segment ratios (0.05). PMMoV outliers and R/IIPs do not align and the laboratory did not analyse CrAssphage. The majority of gene segment ratio outliers occur between March/April 2023 and September 2023, coinciding with generally low SARS concentrations and potentially volatile gene segment ratios, despite values being above the limit of quantification.

### 3.6 Effects of QCP plausibility checks on the WWTP-lab specific data quality

We determined the most suitable QCP for each WWTP-Lab in our dataset. Table 2 shows at how many WWTP-Labs the QCP has been measured and in how many cases it achieved the highest F1-score among all measured QCPs. We found that 140 WWTP-Labs had a QCP with an F1-score greater than 0. The F1-score results for all WWTP-Lab can be found in Supplementary Table S2.

**Table 2:** Results of the F1-score calculation.

QCP	Count	Highest F1	Share
Gene segment ratio	130	40	0.308
Flow	157	40	0.255
CrAssphage	28	5	0.179
pH	151	20	0.132
PMMoV	122	16	0.131
EC	157	17	0.108

Shown are the number of WWTP-Labs measuring the QCP, number and share of WWTP-Lab where the respective QCP achieved the highest F1-score in comparison the other QCP.

Overall, gene segment ratios and Q achieve the highest F1-score more frequently (in 31 and 26% of all WWTP-Labs) than other QCPs but are also more frequently measured. The electrical conductivity has the lowest share of rank 1 F1-scores, followed by pH.

**Figure 8:**
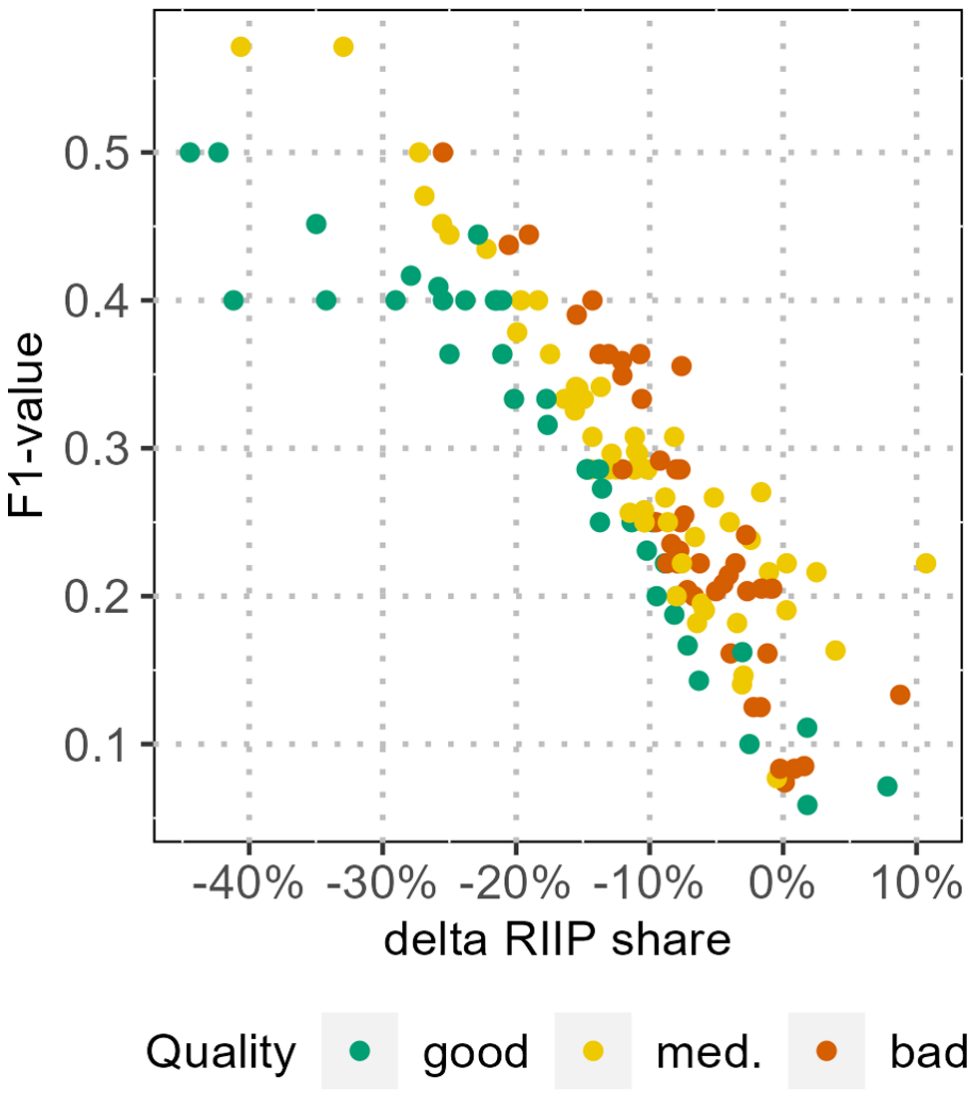
Relative R/IIP share changes due to rank 1 QCP-plausibility check and the F1-score of the respective parameter. The quality category is based on the share of R/IIP. An F1-score of 1 signifies that all outliers in the selected QCP and outliers in R/IIP match, an F1-score of 0, that there is no match.

Figure 8 displays both the relative change of the R/IIP share due to a plausibility check with the QCP with the highest F1-score and the F1-score of the used QCP. For the majority of the WWTPs, the QCP plausibility checks reduce the R/IIP share. The relative R/IIP share decrease is rather small with a median of −10% (Q1 = −16% and Q3 = −5%). So is the F1-score with a median of 0.27 (Q1 = 0.20 and Q3 = 0.36). Nevertheless, apart from 11 WWTP-Labs, our model improves the data quality by removing QCP-based outliers.

The decrease of the R/IIP share of WWTP-Labs with ‘good’ data quality has a median of −15%, while the median for ‘mediocre’ data quality is −11% and ‘bad’ data quality is −7%. Likewise, the rank 1 F1-scores are higher for WWTP-Labs with a better initial trend quality. The medians are 0.29 for ‘good’ and ‘mediocre’ quality and 0.23 for ‘bad’ quality.

## 4 Discussion

### 4.1 Assessing the QCP data

Our dataset consists of approximately 150 WWTPs and reveals an extensive diversity among them. This becomes evident when analysing the variation within single QCP and the varied correlations observed for the QCPs. A precondition for a successful normalisation of SARS concentration data and our plausibility check is that QCPs are relatively constant in wastewater of domestic origin and therefore can be used to approximate its share of the total wastewater. As the flow of domestic wastewater is assumed to be rather constant, we expect a near-linear relationship to the QCPs.

The variation in the wastewater flow is plausible. Most outliers are associated with flows higher than the median flow, whereas there is minimal variation in flows lower than the median flow. WWTPs typically maintain a relatively constant minimum flow, known as ‘dry weather flow’, which largely consists of domestic wastewater, continuously discharged industrial wastewater, and a rather constant groundwater infiltration. Tourism or vacations can lead to a fluctuating population size and therefore have an impact on domestic wastewater volumes. Outliers below the median are likely attributable to transmission errors or failures in the sewer system. Conversely, outliers above the median can be attributed to precipitation, irregular discharges from industries, or major social events such as festivals. The magnitude of these effects is largely dependent on the sewer system, including its type (e.g., combined versus separated sewer systems), its size and expansion, and its topographical location. Furthermore, we have no additional meta data for the sampling days and a varying number of these events might be covered in the dataset of each WWTP. Hence, the direct comparison of flow variability might be misleading.

Our data indicates that the ‘surrogate’ viruses PMMoV and CrAssphage are not universally applicable indicators for the proportion of domestic wastewater. Nevertheless, our data analysis shows that there are few WWTPs where we see a near-linear negative relationship between Q and PMMoV. This is in line with studies, where normalisation with PMMoV could improve SARS data quality for some WWTPs (23) and could not improve the SARS data quality for other WWTPs (32, 33, 34). While the correlation coefficients for electrical conductivity with Q are slightly higher compared to PMMoV and CrAssphage, good correlation coefficients (below −0.75) are observed only in very few WWTPs. Therefore, the hypothesis of a dilution of domestic wastewater with increasing Q cannot be reliably confirmed with PMMoV, CrAssphage, or EC as indicators, this is in line with Nagarkar et al. (28) and Maal-Bared et al. (23). Hence, using a single QCP as a proxy for the share of domestic wastewater is not suitable for all WWTPs and a respective normalisation proves ineffective for our entire dataset. However, normalisation with a single QCP or a uniform combination is commonly suggested in many studies [e.g., (24, 35), or the recommendation of the European Commission for a uniform surveillance approach (36)].

It is evident that highly diverse processes within WWTPs and sewer systems influence both flow and other QCPs. For PMMoV, Kitajima et al. (37) and Hsu et al. (26) present extensive analyses on the PMMoV concentration dynamics in the population, emphasising socio-cultural factors among others. Furthermore, a varying flow indicates different dynamics in the sewer system and together with the catchment size has an impact on the travel and thus degradation time from the source to the WWTP. This effect may enhance non-linear relations between the ‘surrogate’ viruses and Q. Also, EC is not necessarily a good parameter to approximate the share of domestic wastewater, since a mere dilution by stormwater or industrial discharges cannot be assumed. To estimate the proportion of domestic wastewater and to interpret the variations in the QCPs, a better knowledge on the WWTP level would be needed. This includes additional data (e.g., precipitation data or information on industrial discharges) but also expert knowledge. Therefore, we investigated irregularities in the SARS concentration curve at WWTP 73 to evaluate whether the statistical outliers match with properties of the wastewater composition. We noticed a strong phenotypic variability of the wastewater, which on some days showed a strong colouration indicating a drastic change in wastewater composition and a change in the respective water chemistry. This was cross-checked with replicate samples and laboratory errors could be excluded. These changes may have been related to batch-type industrial discharges or other sources of external water but the true cause is unknown. Additional, even more detailed investigations would be necessary to relate these observations to outliers in the SARS concentration curve.

We have identified the correlation between different gene segments as one possible indicator for the reliability of the SARS analysis. This is in accordance with studies evaluating the combination of different gene segments, e.g., Ho et al. (12) who excluded N1 based on insufficient correlation to other gene segments. Furthermore, the gene segment ratio has a direct impact on the SARS concentration curve, where usually mean concentrations of several gene segments are used. In consequence, unsteady curves may not be related to the WWTP, but more to the laboratory analysing the samples. In our dataset, the degree of correlation could not be attributed to specific WWTPs, laboratories, or the chosen gene segments. Hence, we assume that multiple factors have an impact on the gene segment ratios. The sample preparation and analysis (e.g., concentration, extraction, and detection methods) may have an influence on gene segment ratios. In addition, inhibitors have an impact on the PCR-analysis, they may affect the sensitivity of the assay and cause diminished viral recovery and detection (38, 39). The inhibition may affect gene segments differently, so the wastewater composition has an influence on the ratios. The effect of inhibitors may also depend on the chosen laboratory methods.

Taking all the factors mentioned above into consideration, we expect that the variation in the QCPs can be mostly attributed to the variability in and between the sewer systems. Nevertheless, a standardisation of sampling protocols, including PCR inhibition control may improve QCP data and facilitate the comparison between different WWTPs. We also recommend the standardisation of laboratory methods to improve comparability and the correlation between different gene segments. We suggest to set a boundary for WWTP-Lab-specific Pearson correlation coefficient to at least 0.9 for all gene segment combinations to reduce the data spread.

### 4.2 Assessing the SARS data quality

We used outliers in the reproduction rate of the SARS concentration curves to evaluate the SARS concentration data quality for each WWTP-Lab. Instead of correlating wastewater data with clinical data [e.g., (1, 12–15, 28, 32, 40)], this method evaluates the course of the curve itself and we consider our method a reliable proxy for the SARS concentration trend quality as the results align well with visual assessments. Similar methods have been applied in other studies, e.g., Sakarovitch et al. (41) evaluate the distance of each datapoint to a smoothed LOESS curve. Upon analysing the complete dataset of 157 WWTP-Labs, significant variations in the share of outliers among different WWTP-Lab combinations become evident. When SARS concentrations follow a ‘good’ trend (as depicted in Figure 4A) it becomes relatively straightforward to gauge moments of low or high prevalence and discern whether the trend is rising or falling. Retrospectively, outbreaks can be identified clearly. Conversely, for WWTP-Labs we classified as having a ‘bad’ trend (illustrated in Figure 4B), assessing the SARS concentration trend becomes challenging. Thus, the evaluation of trend quality based on the reproduction rate is a valuable method and a good alternative to the correlation with clinical data, especially when clinical data is no longer reliable or susceptible to frequent changes, e.g., through mandatory testing.

The ability to discern between ‘good’ and ‘bad’ data is especially relevant on the local level. Health authorities need recognisable trends to include WBE information in decision-making. Experience from previous projects has shown that public health authorities handled the data differently in the early and late pandemic. In the early pandemic, SARS monitoring was strongly based on clinical data such as the 7-day incidence, and wastewater data was primarily used as an indicator of a potential change in the infection dynamics. For example, repeated high SARS concentrations in wastewater at a site were used as an indicator of local infection events (e.g., large gatherings), which then led to an initial investigation of the local situation or a targeted campaign of rapid testing. As the pandemic progressed, the situation changed to a more holistic view of wastewater data. Time series allowed to assess the expected variance of the data and estimate the reliability of the data in general. This was then used by public health authorities to identify the end of an infection wave (e.g., 2022 for the first waves of Omicron), which was helpful in recommending or not recommending changes in public health policies. However, the collaboration with public health authorities during the pandemic was always linked to a direct information exchange with experts. An automated assessment of the data quality therefore saves valuable resources and supports these experts to give local health authorities further guidance.

To observe national or regional trends, SARS concentration data from several WWTP-Labs can be aggregated. The aggregation of data from various WWTP-Labs is potentially biased as the data was obtained through different sampling and analysis methods. However, as long as the number of different WWTPs is large enough, differences in data quality are averaged out. As the WWTPs are spread across Germany, a good approximation of the national average can be assumed. Our data suggests that trend recognition improves when including a larger number of WWTP-Labs in an aggregated curve, even those with high R/IIP-shares. However, we have demonstrated that the individual data quality reflects in the aggregated curve. Considering only those 33% of WWTP-Labs with lowest R/IIP shares yields an aggregated curve that is very similar to an aggregated curve over all WWTP-Labs. Hence, ensuring good data quality is crucial when setting up cost-efficient WBE monitoring systems and choosing WWTP-Labs for aggregated curves. For Bangkok, Sangsanont et al. (42) have shown that the 4 largest WWTPs are enough to derive reliable trends for all 19 available WWTPs.

### 4.3 Assessing the effects of QCP data plausibility checks

We analysed the effect of data plausibility checks with QCPs by comparing outliers in QCPs with R/IIPs in the SARS curve and quantifying the fit with the F1-score. The QCP with the highest F1-score differs from WWTP to WWTP. Yet in general, gene segment ratios and the flow outperform other QCPs. Outliers in gene segment ratios may indicate errors in the analysis process, with a direct impact on the resulting SARS concentration while outliers in the flow may indicate extreme events such as heavy rain. However, both effects cannot be observed for all datapoints within a WWTP-Lab with a good F1-score for gene segment ratio or the flow, nor regularly for all WWTP-Labs. The F1-scores of the top-ranked parameters are generally low, suggesting that the trend improvements from QCP-plausibility checks are small. This is also confirmed by the relatively low improvement of the R/IIP-share after outlier removal. However, our model is more successful in removing R/IIPs for WWTP-Labs with a good initial quality category, hence with a low R/IIP share in the unprocessed data.

Low F1-scores indicate that only few potential outliers through QCP checks are identified and that the confidence, that these outliers are actual outliers is low. As a practical implication for decision-making processes, we recommend to consider the QCP with the highest F1-score and only if this score is relatively high. However, whether an F1-score is high enough and the impact of our model need to be assessed by experts for each individual WWTP.

Regarding the outcomes of our model, it becomes evident that the majority of implausible datapoints in the SARS concentration curve cannot be attributed to irregular events. One possible explanation is that these events do not reflect in the available QCPs, for example the irregular discharge of PCR-inhibiting substances which are not covered in our data. With respect to the temporal resolution, QCPs are usually daily averages but they may affect the SARS concentration completely different. A long-lasting heavy stormwater event that dilutes wastewater during the daily SARS concentration peak has a larger impact on the daily SARS concentration compared to a stormwater event that takes place at night when the SARS concentration is typically much lower. The model treats both cases the same, with different success for the plausibility check. Data with a higher temporal resolution, as well as specific sewer and wastewater treatment system knowledge are mandatory to assess if the influence of QCP variations on the SARS concentration is plausible. Furthermore, the variation in the SARS concentration data may be caused by the applied sampling or analysis methods. The resulting uncertainties have been investigated by many studies [e.g., (21, 43, 44)], yet their systematic integration in our model is beyond the scope of this study.

In contrast to normalisation methods, our model reduces the number of values in a dataset and the success of our model is related to the boundaries for identifying outliers in both, QCPs and R/IIPs. The outlier removal functions hinge on the chosen scale (set to 1.5 of the IQR) and whether the entire dataset should be used to identify outliers or just WWTP and laboratory-specific data. These parameters need to be adapted carefully to find a good balance between improving the trend quality and preventing information loss in the dataset. An advanced model which optimises the boundaries on a WWTP-Lab level may improve data quality even further. Hence, the selection of both statistical parameters and the QCPs becomes an optimization problem for each WWTP, laboratory, and period. Instead of considering only statistical relations, additional information can be key to interpret data on the WWTP level. Data on sewer systems, industrial discharges, population dynamics, or precipitation can help to choose suitable QCPs and to set sensible boundaries in the model. Collecting additional data and including them in the model may improve its success. In addition, there are alternatives to treat QCP outliers. For example, instead of excluding them from the SARS concentration curve, the specific SARS concentration can be given less weight by, e.g., averaging them with previous values when calculating the trend.

## 5 Conclusion

In this study, we manipulated WBE data to improve the recognition of SARS concentration trends and therefore enhance its value for health authorities in decision-making processes. We assessed whether the additional information provided by quality control parameters (QCP) is useful to assess the plausibility of SARS concentration data. The analysed dataset includes data from around 150 participating WWTPs across all Germany.

Our newly introduced quality indicator, the R/IIP share, is suitable to evaluate and compare the reliability of SARS trends on the WWTP level and is therefore a good addition or alternative to the correlation with clinical data. We set up a model that automatically flags implausible SARS concentration datapoints based on outliers in QCPs. It becomes evident, that the most successful QCP to identify and remove R/IIPs is different for each WWTP, however, flow and gene segment ratio outperform other QCPs, such as electrical conductivity or the ‘surrogate viruses’ PMMoV and CrAssphage.

Overall, our model enhances trend quality, although only slightly. By individually optimising the boundaries of the model, its success could be enhanced for each WWTP. For this, additional expert knowledge of the properties of the local wastewater and sewer system are pivotal.

The results of our data plausibility checks emphasise the need for reliable data when establishing a nationwide WBE system, including a high-quality sample analysis. Hence, WWTPs and laboratories must be chosen carefully before including them in the dataset. Our model enhances the value of WBE SARS concentrations data for public health authorities. It can be used to assess and improve the reliability of SARS concentration trends when using WBE data for decision-making processes. Furthermore, it is a helpful tool when choosing WWTPs for cost-efficient WBE systems.

## Data Availability

The datasets presented in this study can be found in online repositories. The names of the repository/repositories and accession number(s) can be found at: https://github.com/nathanobermaier/WBE_SARS_data_analysis/releases/latest.
