# Supplementary material for: Wastewater-based epidemiology: deriving a SARS-CoV-2 data validation method to assess data quality and to improve trend recognition
Source: Front Public Health. 2024 Dec 12;12:1497100. doi: 10.3389/fpubh.2024.1497100 (PMC11674844; doi:10.3389/fpubh.2024.1497100)
Supplement: Supplementary file 1 [file Data_Sheet_1.docx]

Supplementary Material

**Wastewater-based Epidemiology: Deriving a SARS-CoV-2 Data Validation Method to Assess Data Quality and to Improve Trend Recognition**

# Code & Input Data

Input data and R code can be found under the following link:

https://github.com/nathanobermaier/WBE_SARS_data_analysis/releases/latest

# Sampling, Sample Preparation, Detection, Automated Data Checks

Wastewater samples are taken twice a week at each participating WWTP and cover sampling periods from Monday to Tuesday morning and Wednesday to Thursday morning. The samples are taken at the inflow of the WWTP, preferably after the grit chamber. An automatic sampler is used, providing 24-hour composite samples, which are either time proportional or volume proportional to the inflow. During the sampling period, a constant temperature of 5 ± 3°C is ensured within the sampler. The composite sample is homogenized thoroughly before transferring 1 L of the sample into a transport container. Directly after sampling, the sample is sent to the respective laboratory, maintaining a temperature of 5°C.

Laboratories are required to develop their own sample preparation and analysis protocol, following some general instructions. After arrival in the laboratory, samples must be analysed as quick as possible. First, a homogenization is carried out by shaking the sample for 15 minutes. To concentrate virus particles, a variety of methods can be used, most commonly applied are PEG (polyethylene glycol)-precipitation, filtration, or centrifugation. The different methods require different input volumes ranging between 40 ml and 1 L. After concentration, the viral RNA is extracted, most commonly by organic extraction. The RNA is transcribed into cDNA by reverse transcription. Amplification and quantification of SARS gene segments must be carried out by either qPCR (quantitative polymerase chain reaction) or dPCR (digital PCR). The target areas of the SARS gene can be either the nucleocapsid region (N1, N2) the gene regions for the envelope protein (E), the polymerase region (RdRp), the spike protein (S), and the open reading frame region (ORF). For PCR analysis, positive and negative controls must be included in parallel. qPCR measurements are carried out as triplicates and all results are reported as gene copies/L.

Automated checks are run on several parameters before the data upload and guarantee a minimum data quality. All data must be assigned to a WWTP, a laboratory, have a sample date, report a sampling period of approx. 24 hours, Q, and at least two different gene segments. In addition, defined ranges for some of the parameters are set in the database to identify typos and obvious mistakes (pH = 0-14, Q = 5-17000 L/s, EC = 250-5000 µS/cm, concentration for each SARS-CoV-2 gene segment, PMMoV and CrA = 1- 1010 gc/L).

# Variation in Q, pH, EC, PMMoV and CrA among the different WWTP

There is a large variability between the different participating WWTP. This is reflected in the QCP daily inflow, pH, electrical conductivity, PMMoV and CrAssphage. Figure S1 to Figure S5 show the variability of the parameter within each WWTP. To improve the comparison, all parameters are normalised with their respective median for the WWTP.


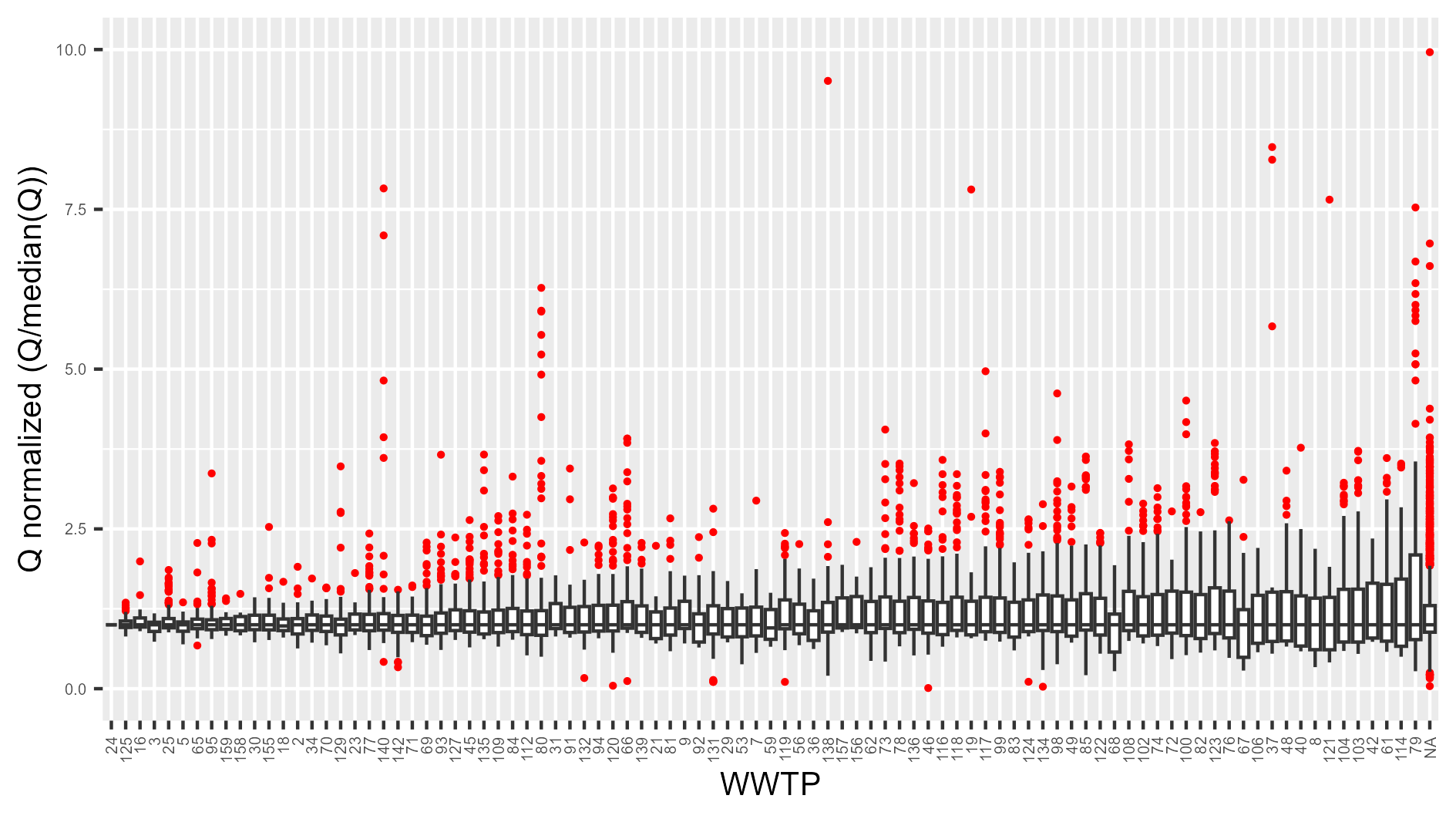
 Figure S1: Variability of inflow: Q normalised for all WWTP


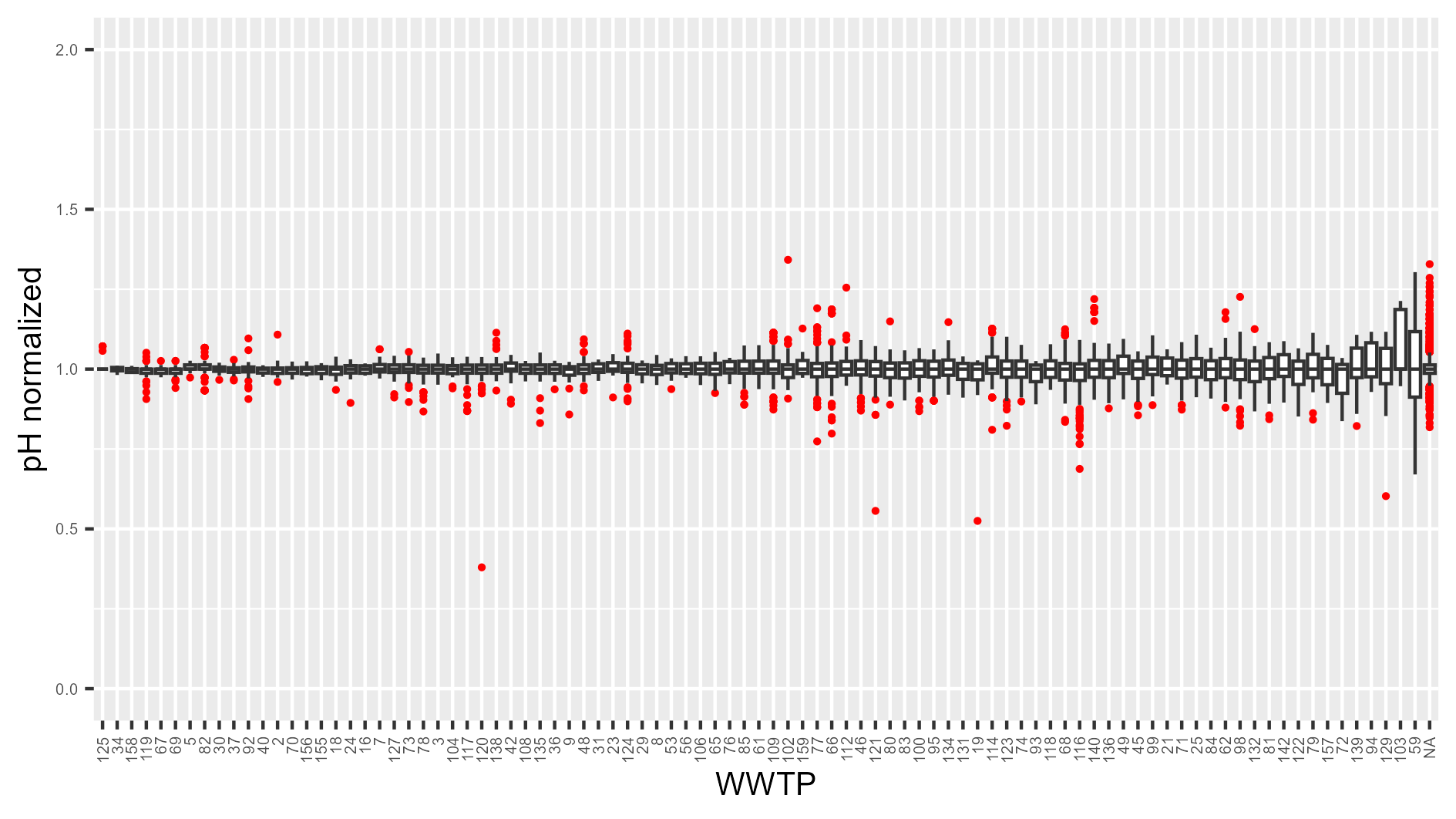


Figure S2: Variability of pH: pH normalised for all WWTP


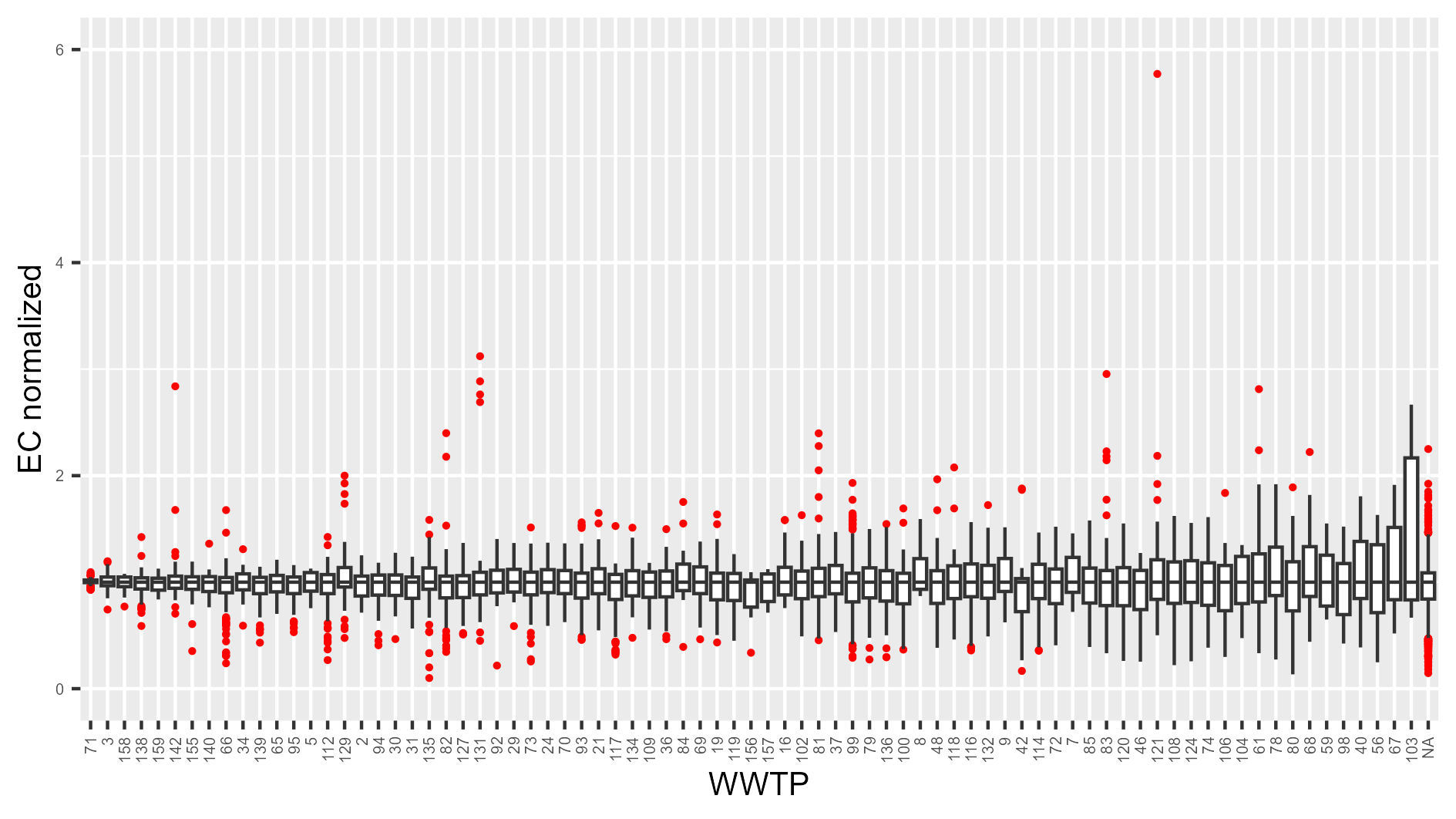
 Figure S3: Variability of EC: EC normalised for all WWTP


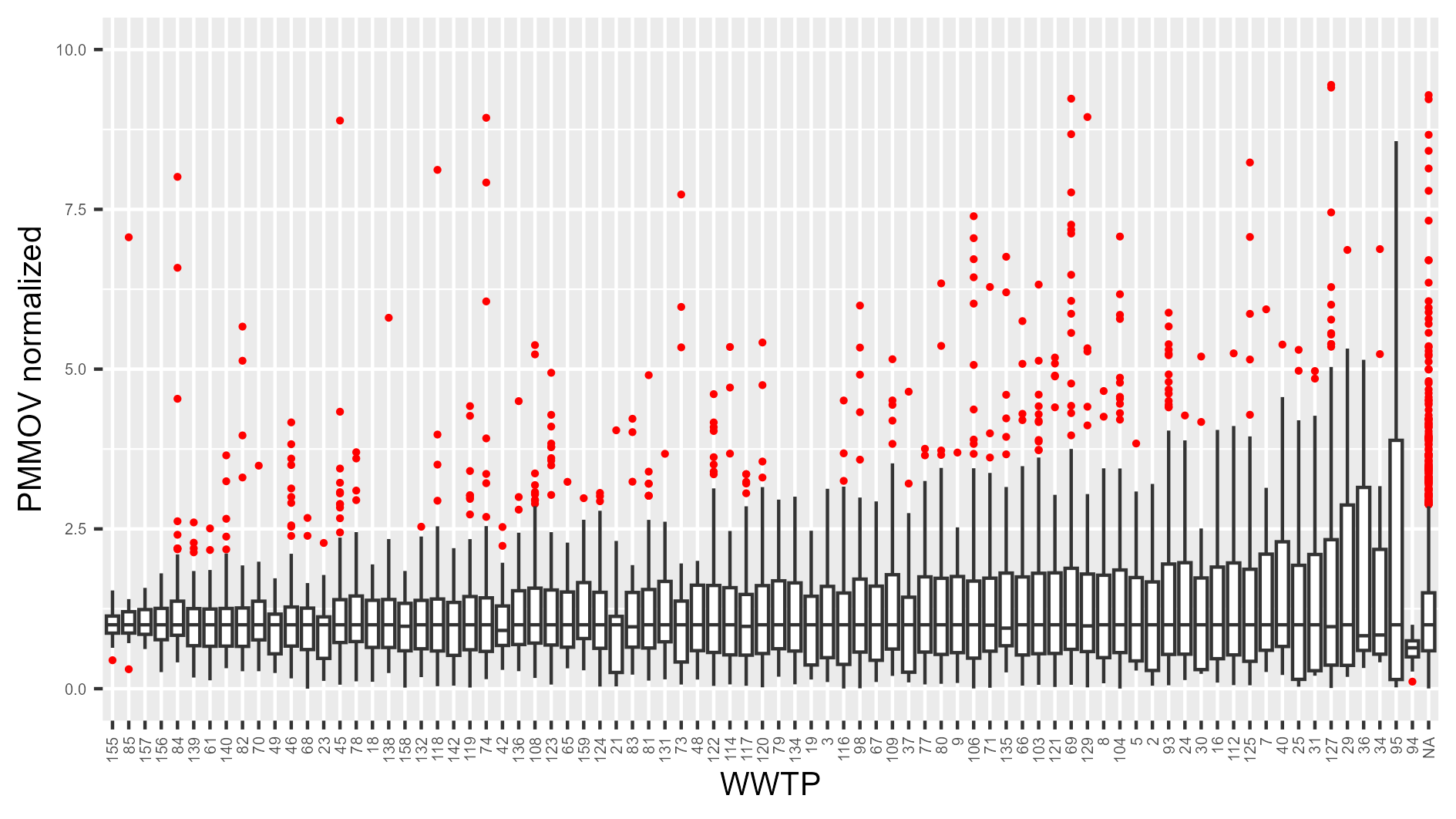
 Figure S4: Variability of PMMoV, PMMoV normalised for all WWTP


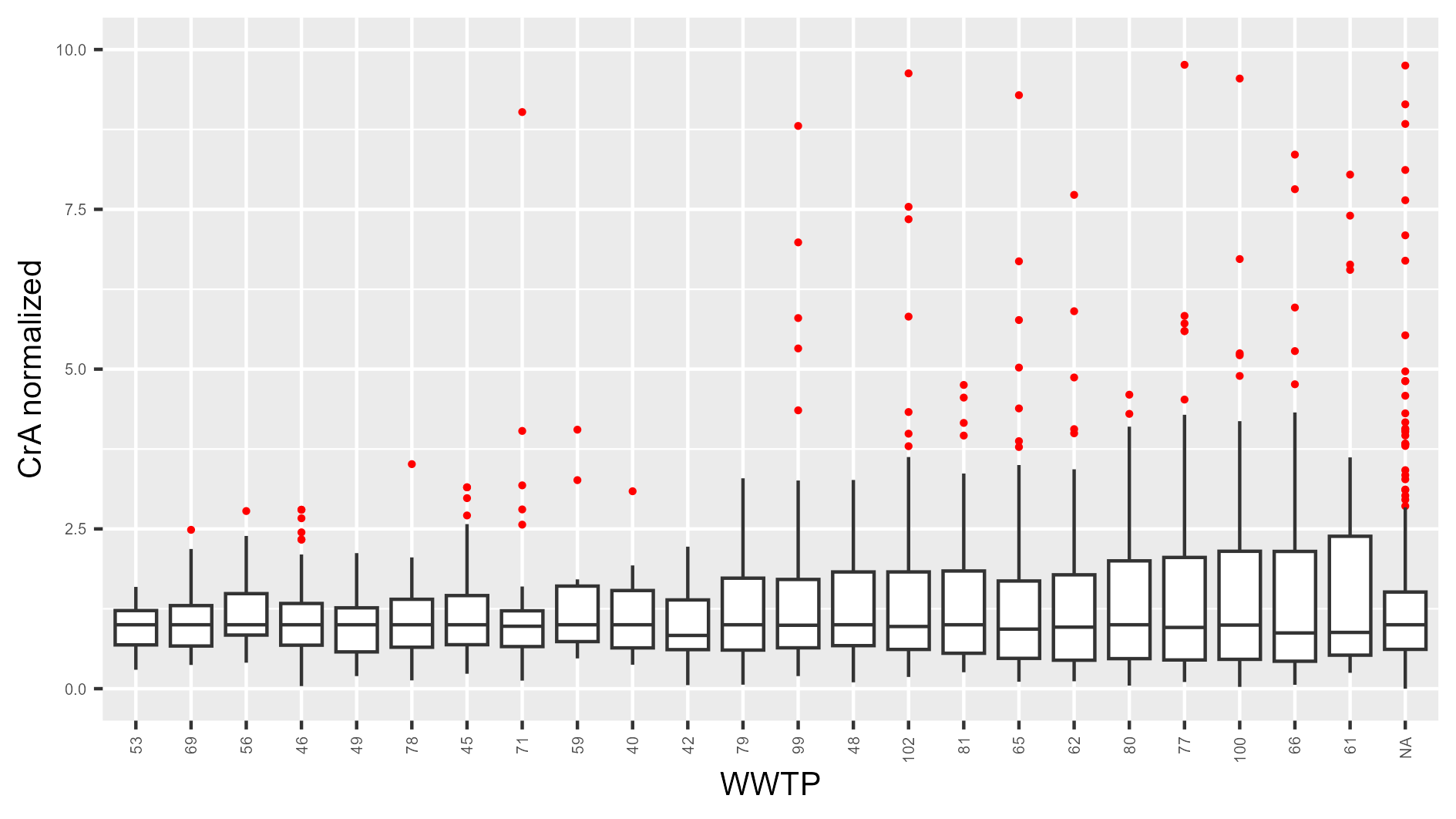
 Figure S5: Variability of CrAssphage, CrA normalised for all WWTP

# Gene segment ratios for all WWTP/Lab

The ratios between the different gene segments indicate high variability. Table S1 shows the gene segment combinations for all WWTP and laboratory combinations. Displayed are the median of the respective gene segment ratios, the number of samples and the Pearson correlation coefficient of the respective gene segments.

Table S1: Gene segment ratios. Displayed are the median of the ratio, the number of samples and the Pearson correlation coefficient for each WWTP, laboratory and analysed gene segment combination.

| **WWTP** | **Laboratory** | **Gene segments** | **Median** | **No. Samples** | **Correlation coefficient (Pearson)** |
| --- | --- | --- | --- | --- | --- |
| 1 | 1 | E N2 | 1,37 | 21 | 0,894 |
| 1 | 6 | E ORF | 1,28 | 101 | 0,967 |
| 1 | 6 | E RdRp | 1,02 | 99 | 0,944 |
| 1 | 6 | ORF RdRp | 0,82 | 99 | 0,934 |
| 1 | 26 | E N2 | 0,71 | 54 | 0,956 |
| 2 | 1 | E N2 | 1,16 | 24 | 0,731 |
| 3 | 1 | E N2 | 1,47 | 21 | 0,918 |
| 4 | 1 | E N2 | 1,33 | 24 | 0,897 |
| 5 | 1 | E N2 | 1,29 | 21 | 0,954 |
| 6 | 1 | E N2 | 1,09 | 21 | 0,906 |
| 7 | 1 | E N2 | 1,29 | 24 | 0,787 |
| 8 | 1 | E N2 | 1,26 | 25 | 0,841 |
| 9 | 1 | E N2 | 1,3 | 25 | 0,919 |
| 10 | 1 | E N2 | 1,56 | 25 | 0,899 |
| 11 | 19 | E ORF | 1,27 | 87 | 0,993 |
| 11 | 19 | ORF RdRp | 0,77 | 89 | 0,972 |
| 11 | 19 | E RdRp | 1 | 89 | 0,969 |
| 11 | 26 | E N2 | 0,74 | 32 | 0,994 |
| 16 | 1 | E N2 | 1,42 | 23 | 0,918 |
| 17 | 1 | E N2 | 1,1 | 22 | 0,903 |
| 18 | 1 | E N2 | 1,09 | 20 | 0,924 |
| 19 | 1 | E N2 | 1,29 | 25 | 0,919 |
| 20 | 21 | E N2 | 1,06 | 88 | 0,838 |
| 21 | 1 | E N2 | 1,28 | 24 | 0,938 |
| 22 | 1 | E N2 | 1,18 | 23 | 0,906 |
| 23 | 1 | E N2 | 1,14 | 16 | 0,881 |
| 24 | 1 | E N2 | 1,29 | 25 | 0,871 |
| 25 | 2 | N1 N2 | 2 | 112 | 0,969 |
| 25 | 13 | E ORF | 0,53 | 33 | 0,873 |
| 25 | 13 | E N1 | 0,78 | 87 | 0,462 |
| 26 | 2 | N1 N2 | 2,25 | 117 | 0,982 |
| 26 | 13 | N1 N2 | 2,15 | 34 | 0,948 |
| 26 | 13 | E N1 | 0,77 | 23 | 0,878 |
| 27 | 2 | N1 N2 | 2,25 | 113 | 0,987 |
| 27 | 13 | E N1 | 0,58 | 20 | 0,935 |
| 29 | 3 | N1 N2 | 0,56 | 15 | 0,983 |
| 30 | 3 | N1 N2 | 0,63 | 19 | 0,928 |
| 31 | 3 | N1 N2 | 0,68 | 17 | 0,995 |
| 34 | 3 | N1 N2 | 0,58 | 16 | 0,984 |
| 35 | 3 | N1 N2 | 0,65 | 21 | 0,945 |
| 35 | 11 | E N2 | 0,74 | 108 | 0,848 |
| 36 | 3 | N1 N2 | 0,63 | 21 | 0,96 |
| 37 | 3 | N1 N2 | 0,63 | 16 | 0,983 |
| 38 | 3 | N1 N2 | 0,55 | 18 | 0,947 |
| 40 | 4 | E N1 | 0,96 | 27 | 0,972 |
| 40 | 7 | N1 RdRp | 0,75 | 19 | 0,792 |
| 40 | 7 | N1 N2 | 2 | 15 | 0,007 |
| 41 | 4 | E N1 | 0,96 | 23 | 0,974 |
| 41 | 7 | N1 RdRp | 0,62 | 15 | 0,859 |
| 42 | 6 | E RdRp | 1,01 | 66 | 0,991 |
| 42 | 6 | E ORF | 1,29 | 67 | 0,981 |
| 42 | 6 | ORF RdRp | 0,75 | 67 | 0,979 |
| 43 | 5 | E N2 | 0,84 | 119 | 0,719 |
| 43 | 5 | N1 N2 | 0,56 | 22 | 0,649 |
| 44 | 5 | N1 N2 | 0,39 | 17 | 0,873 |
| 44 | 5 | E N2 | 0,83 | 103 | 0,766 |
| 45 | 6 | N1 RdRp | 0,9 | 24 | 0,977 |
| 45 | 6 | E RdRp | 0,96 | 166 | 0,975 |
| 45 | 6 | E N1 | 1,06 | 24 | 0,973 |
| 45 | 6 | E ORF | 1,18 | 169 | 0,973 |
| 45 | 6 | N1 ORF | 1,2 | 24 | 0,962 |
| 45 | 6 | ORF RdRp | 0,82 | 167 | 0,962 |
| 46 | 6 | N1 RdRp | 1,23 | 25 | 0,986 |
| 46 | 6 | E N1 | 0,8 | 25 | 0,983 |
| 46 | 6 | E ORF | 1,29 | 203 | 0,979 |
| 46 | 6 | N1 ORF | 1,56 | 25 | 0,974 |
| 46 | 6 | E RdRp | 0,99 | 201 | 0,969 |
| 46 | 6 | ORF RdRp | 0,77 | 200 | 0,968 |
| 47 | 9 | E RdRp | 1,01 | 35 | 0,993 |
| 47 | 9 | E ORF | 1,16 | 35 | 0,986 |
| 47 | 9 | ORF RdRp | 0,86 | 35 | 0,985 |
| 47 | 9 | N1 ORF | 1,36 | 35 | 0,952 |
| 47 | 9 | E N1 | 0,83 | 35 | 0,935 |
| 47 | 9 | N1 RdRp | 1,15 | 35 | 0,929 |
| 48 | 6 | ORF RdRp | 0,81 | 27 | 0,979 |
| 48 | 6 | E RdRp | 1,09 | 27 | 0,959 |
| 48 | 6 | E ORF | 1,43 | 28 | 0,947 |
| 48 | 8 | E RdRp | 1,01 | 80 | 0,995 |
| 48 | 8 | E ORF | 1,17 | 79 | 0,994 |
| 48 | 8 | ORF RdRp | 0,86 | 80 | 0,994 |
| 48 | 8 | N1 RdRp | 0,93 | 54 | 0,986 |
| 48 | 8 | E N1 | 1,03 | 55 | 0,986 |
| 48 | 8 | N1 ORF | 1,1 | 55 | 0,984 |
| 49 | 8 | E ORF | 1,15 | 95 | 0,995 |
| 49 | 8 | E RdRp | 1 | 97 | 0,994 |
| 49 | 8 | ORF RdRp | 0,89 | 97 | 0,991 |
| 49 | 8 | N1 ORF | 1,15 | 60 | 0,983 |
| 49 | 8 | N1 RdRp | 1,08 | 62 | 0,981 |
| 49 | 8 | E N1 | 0,95 | 62 | 0,978 |
| 53 | 7 | N1 RdRp | 0,85 | 15 | 0,784 |
| 56 | 7 | N2 RdRp | 0,5 | 15 | 0,948 |
| 56 | 7 | N1 RdRp | 1,36 | 20 | 0,513 |
| 56 | 7 | N1 N2 | 2,12 | 15 | 0,45 |
| 59 | 7 | N1 RdRp | 0,7 | 17 | 0,942 |
| 61 | 7 | N3 RdRp | 1 | 36 | 0,966 |
| 61 | 7 | N1 N2 | 2,21 | 44 | 0,785 |
| 61 | 7 | N2 RdRp | 0,93 | 45 | 0,718 |
| 61 | 7 | N1 RdRp | 1,2 | 43 | 0,608 |
| 61 | 8 | ORF RdRp | 0,9 | 84 | 0,997 |
| 61 | 8 | E RdRp | 1,01 | 85 | 0,996 |
| 61 | 8 | N1 RdRp | 0,92 | 60 | 0,995 |
| 61 | 8 | E N1 | 1,03 | 61 | 0,995 |
| 61 | 8 | E ORF | 1,11 | 86 | 0,995 |
| 61 | 8 | N1 ORF | 1,01 | 60 | 0,993 |
| 62 | 7 | N1 N2 | 1,86 | 76 | 0,846 |
| 62 | 7 | N1 RdRp | 1,44 | 76 | 0,802 |
| 62 | 7 | N2 RdRp | 0,98 | 72 | 0,778 |
| 62 | 9 | ORF RdRp | 0,9 | 72 | 0,995 |
| 62 | 9 | E RdRp | 0,99 | 64 | 0,992 |
| 62 | 9 | E ORF | 1,11 | 66 | 0,992 |
| 62 | 9 | E N1 | 0,86 | 66 | 0,982 |
| 62 | 9 | N1 ORF | 1,23 | 74 | 0,97 |
| 62 | 9 | N1 RdRp | 1,15 | 71 | 0,966 |
| 63 | 7 | N1 N2 | 1,69 | 87 | 0,774 |
| 63 | 7 | N1 RdRp | 1,51 | 87 | 0,769 |
| 63 | 7 | N2 RdRp | 0,8 | 86 | 0,696 |
| 63 | 9 | N1 ORF | 1,19 | 77 | 0,99 |
| 63 | 9 | N1 RdRp | 1,09 | 78 | 0,985 |
| 63 | 9 | ORF RdRp | 0,9 | 78 | 0,985 |
| 63 | 9 | E RdRp | 0,98 | 70 | 0,981 |
| 63 | 9 | E ORF | 1,09 | 68 | 0,978 |
| 63 | 9 | E N1 | 0,89 | 68 | 0,971 |
| 64 | 7 | N2 RdRp | 0,66 | 68 | 0,852 |
| 64 | 7 | N1 N2 | 2,22 | 67 | 0,814 |
| 64 | 7 | N1 RdRp | 1,33 | 66 | 0,652 |
| 64 | 8 | E RdRp | 1 | 71 | 0,993 |
| 64 | 8 | E ORF | 1,05 | 70 | 0,986 |
| 64 | 8 | ORF RdRp | 0,95 | 71 | 0,981 |
| 64 | 8 | E N1 | 0,83 | 45 | 0,976 |
| 64 | 8 | N1 ORF | 1,28 | 45 | 0,974 |
| 64 | 8 | N1 RdRp | 1,2 | 47 | 0,966 |
| 65 | 7 | N1 N2 | 3,21 | 77 | 0,814 |
| 65 | 7 | N1 RdRp | 1,21 | 77 | 0,491 |
| 65 | 7 | N2 RdRp | 0,35 | 79 | 0,321 |
| 65 | 26 | E N2 | 0,73 | 34 | 0,988 |
| 66 | 7 | N1 N2 | 1,64 | 85 | 0,768 |
| 66 | 7 | N1 RdRp | 1,11 | 86 | 0,627 |
| 66 | 7 | N2 RdRp | 0,74 | 86 | 0,541 |
| 66 | 20 | N1 N2 | 0,65 | 81 | 0,94 |
| 67 | 21 | E N1 | 0,7 | 24 | 0,701 |
| 68 | 21 | E N1 | 0,26 | 30 | 0,718 |
| 69 | 7 | N1 RdRp | 0,63 | 20 | 0,68 |
| 69 | 21 | E N2 | 0,99 | 104 | 0,887 |
| 70 | 21 | E N1 | 0,66 | 24 | 0,858 |
| 71 | 7 | N1 N2 | 3,91 | 43 | 0,87 |
| 71 | 7 | N2 RdRp | 0,33 | 46 | 0,68 |
| 71 | 7 | N1 RdRp | 1,09 | 42 | 0,653 |
| 71 | 22 | N1 N2 | 0,88 | 31 | 0,88 |
| 71 | 22 | E N2 | 0,38 | 34 | 0,796 |
| 72 | 8 | E RdRp | 1,01 | 20 | 0,998 |
| 72 | 8 | E ORF | 1,14 | 20 | 0,994 |
| 72 | 8 | ORF RdRp | 0,85 | 21 | 0,991 |
| 73 | 10 | E N2 | 0,74 | 96 | 0,979 |
| 73 | 10 | E RdRp | 0,71 | 59 | 0,953 |
| 73 | 10 | N2 ORF | 2,26 | 99 | 0,952 |
| 73 | 10 | N2 RdRp | 0,79 | 59 | 0,936 |
| 73 | 10 | E ORF | 1,62 | 99 | 0,925 |
| 73 | 10 | ORF RdRp | 0,32 | 61 | 0,899 |
| 74 | 8 | E RdRp | 1 | 83 | 0,993 |
| 74 | 8 | N1 ORF | 1,09 | 59 | 0,992 |
| 74 | 8 | ORF RdRp | 0,88 | 84 | 0,992 |
| 74 | 8 | E ORF | 1,13 | 85 | 0,992 |
| 74 | 8 | N1 RdRp | 0,98 | 60 | 0,991 |
| 74 | 8 | E N1 | 0,98 | 158 | 0,981 |
| 75 | 8 | E RdRp | 1,01 | 105 | 0,996 |
| 75 | 8 | E ORF | 1,09 | 103 | 0,995 |
| 75 | 8 | ORF RdRp | 0,91 | 104 | 0,995 |
| 75 | 8 | N1 ORF | 1,08 | 76 | 0,988 |
| 75 | 8 | N1 RdRp | 1 | 78 | 0,983 |
| 75 | 8 | E N2 | 0,64 | 15 | 0,98 |
| 75 | 8 | E N1 | 0,95 | 77 | 0,98 |
| 75 | 8 | N2 ORF | 1,55 | 15 | 0,951 |
| 75 | 8 | N2 RdRp | 1,63 | 15 | 0,934 |
| 75 | 8 | N1 N2 | 0,79 | 15 | 0,929 |
| 76 | 8 | E RdRp | 1,03 | 23 | 0,995 |
| 76 | 8 | ORF RdRp | 0,85 | 23 | 0,992 |
| 76 | 8 | N1 ORF | 1,36 | 22 | 0,985 |
| 76 | 8 | E ORF | 1,22 | 23 | 0,985 |
| 76 | 8 | N1 RdRp | 1,1 | 22 | 0,982 |
| 76 | 8 | E N1 | 0,94 | 22 | 0,981 |
| 77 | 8 | E RdRp | 0,97 | 78 | 0,993 |
| 77 | 8 | ORF RdRp | 0,91 | 78 | 0,988 |
| 77 | 8 | E ORF | 1,09 | 75 | 0,98 |
| 77 | 8 | N1 RdRp | 1,18 | 50 | 0,968 |
| 77 | 8 | E N1 | 0,83 | 50 | 0,965 |
| 77 | 8 | N1 ORF | 1,31 | 49 | 0,959 |
| 77 | 10 | E ORF | 1,18 | 92 | 0,988 |
| 77 | 10 | E RdRp | 0,95 | 92 | 0,982 |
| 77 | 10 | ORF RdRp | 0,77 | 94 | 0,982 |
| 78 | 8 | E RdRp | 0,99 | 71 | 0,997 |
| 78 | 8 | N1 ORF | 1,09 | 46 | 0,994 |
| 78 | 8 | ORF RdRp | 0,92 | 74 | 0,994 |
| 78 | 8 | N1 RdRp | 0,99 | 46 | 0,993 |
| 78 | 8 | E ORF | 1,1 | 73 | 0,992 |
| 78 | 8 | E N1 | 1,02 | 46 | 0,99 |
| 78 | 10 | E ORF | 1,21 | 84 | 0,99 |
| 78 | 10 | E RdRp | 1,02 | 85 | 0,984 |
| 78 | 10 | ORF RdRp | 0,86 | 86 | 0,981 |
| 79 | 8 | E RdRp | 0,98 | 36 | 0,997 |
| 79 | 8 | N1 ORF | 1,1 | 22 | 0,988 |
| 79 | 8 | ORF RdRp | 0,88 | 36 | 0,987 |
| 79 | 8 | N1 RdRp | 0,99 | 21 | 0,982 |
| 79 | 8 | E ORF | 1,09 | 36 | 0,981 |
| 79 | 8 | E N1 | 1,1 | 22 | 0,968 |
| 79 | 10 | ORF RdRp | 0,84 | 57 | 0,993 |
| 79 | 10 | E ORF | 1,24 | 59 | 0,992 |
| 79 | 10 | E RdRp | 1,02 | 57 | 0,987 |
| 80 | 8 | E RdRp | 0,94 | 21 | 0,998 |
| 80 | 8 | E ORF | 1,12 | 20 | 0,997 |
| 80 | 8 | ORF RdRp | 0,88 | 20 | 0,997 |
| 80 | 8 | N1 ORF | 1,19 | 15 | 0,981 |
| 80 | 8 | N1 RdRp | 1,08 | 16 | 0,977 |
| 80 | 8 | E N1 | 0,9 | 16 | 0,974 |
| 80 | 10 | E ORF | 1,26 | 98 | 0,992 |
| 80 | 10 | E RdRp | 0,98 | 98 | 0,985 |
| 80 | 10 | ORF RdRp | 0,79 | 97 | 0,98 |
| 81 | 8 | E RdRp | 0,98 | 22 | 0,995 |
| 81 | 8 | N1 ORF | 0,98 | 21 | 0,991 |
| 81 | 8 | ORF RdRp | 0,86 | 22 | 0,991 |
| 81 | 8 | E ORF | 1,14 | 23 | 0,983 |
| 81 | 8 | N1 RdRp | 0,84 | 20 | 0,982 |
| 81 | 8 | E N1 | 1,2 | 21 | 0,977 |
| 81 | 10 | E ORF | 1,27 | 75 | 0,982 |
| 81 | 10 | E RdRp | 1,08 | 73 | 0,972 |
| 81 | 10 | ORF RdRp | 0,81 | 74 | 0,965 |
| 82 | 10 | E N2 | 0,69 | 110 | 0,955 |
| 82 | 10 | N2 RdRp | 0,99 | 58 | 0,944 |
| 82 | 10 | E RdRp | 0,76 | 64 | 0,939 |
| 82 | 10 | N2 ORF | 2,23 | 107 | 0,931 |
| 82 | 10 | ORF RdRp | 0,45 | 65 | 0,913 |
| 82 | 10 | E ORF | 1,5 | 112 | 0,778 |
| 83 | 10 | E N2 | 0,71 | 89 | 0,975 |
| 83 | 10 | E RdRp | 0,7 | 51 | 0,953 |
| 83 | 10 | N2 ORF | 2,27 | 90 | 0,938 |
| 83 | 10 | N2 RdRp | 0,96 | 51 | 0,936 |
| 83 | 10 | E ORF | 1,43 | 92 | 0,935 |
| 83 | 10 | ORF RdRp | 0,36 | 52 | 0,929 |
| 84 | 10 | E N2 | 0,67 | 168 | 0,983 |
| 84 | 10 | E RdRp | 0,75 | 65 | 0,961 |
| 84 | 10 | N2 RdRp | 1 | 62 | 0,94 |
| 84 | 10 | ORF RdRp | 0,5 | 66 | 0,934 |
| 84 | 10 | N2 ORF | 1,85 | 171 | 0,927 |
| 84 | 10 | E ORF | 1,18 | 175 | 0,918 |
| 85 | 10 | E N2 | 0,72 | 87 | 0,989 |
| 85 | 10 | E RdRp | 0,7 | 40 | 0,973 |
| 85 | 10 | N2 RdRp | 0,86 | 38 | 0,969 |
| 85 | 10 | N2 ORF | 2,21 | 85 | 0,961 |
| 85 | 10 | E ORF | 1,42 | 89 | 0,943 |
| 85 | 10 | ORF RdRp | 0,36 | 41 | 0,905 |
| 86 | 10 | E N2 | 0,68 | 147 | 0,981 |
| 86 | 10 | E RdRp | 0,7 | 44 | 0,975 |
| 86 | 10 | E ORF | 1,16 | 153 | 0,956 |
| 86 | 10 | N2 RdRp | 0,9 | 43 | 0,949 |
| 86 | 10 | N2 ORF | 1,75 | 151 | 0,938 |
| 86 | 10 | ORF RdRp | 0,42 | 46 | 0,936 |
| 87 | 9 | E RdRp | 1 | 33 | 0,994 |
| 87 | 9 | E ORF | 1,19 | 33 | 0,989 |
| 87 | 9 | ORF RdRp | 0,87 | 33 | 0,989 |
| 87 | 9 | N1 RdRp | 1,2 | 31 | 0,97 |
| 87 | 9 | N1 ORF | 1,37 | 31 | 0,962 |
| 87 | 9 | E N1 | 0,86 | 31 | 0,961 |
| 89 | 9 | E RdRp | 1,01 | 31 | 0,977 |
| 89 | 9 | ORF RdRp | 0,89 | 31 | 0,967 |
| 89 | 9 | N1 ORF | 1,29 | 30 | 0,946 |
| 89 | 9 | E ORF | 1,14 | 31 | 0,943 |
| 89 | 9 | N1 RdRp | 1,1 | 30 | 0,934 |
| 89 | 9 | E N1 | 0,92 | 30 | 0,925 |
| 90 | 10 | E N2 | 0,67 | 191 | 0,975 |
| 90 | 10 | E RdRp | 0,71 | 67 | 0,973 |
| 90 | 10 | N2 ORF | 1,99 | 183 | 0,965 |
| 90 | 10 | N2 RdRp | 0,91 | 67 | 0,947 |
| 90 | 10 | E ORF | 1,28 | 187 | 0,933 |
| 90 | 10 | ORF RdRp | 0,43 | 67 | 0,899 |
| 91 | 9 | E RdRp | 1,01 | 30 | 0,994 |
| 91 | 9 | E ORF | 1,13 | 30 | 0,993 |
| 91 | 9 | ORF RdRp | 0,87 | 30 | 0,992 |
| 91 | 9 | N1 ORF | 1,24 | 28 | 0,989 |
| 91 | 9 | N1 RdRp | 1,1 | 28 | 0,984 |
| 91 | 9 | E N1 | 0,88 | 28 | 0,981 |
| 92 | 9 | E RdRp | 1,01 | 31 | 0,996 |
| 92 | 9 | ORF RdRp | 0,89 | 31 | 0,988 |
| 92 | 9 | E ORF | 1,14 | 31 | 0,987 |
| 92 | 9 | N1 RdRp | 1,06 | 30 | 0,981 |
| 92 | 9 | E N1 | 0,93 | 30 | 0,978 |
| 92 | 9 | N1 ORF | 1,24 | 30 | 0,971 |
| 93 | 11 | E N2 | 0,82 | 116 | 0,838 |
| 93 | 26 | E N2 | 0,73 | 44 | 0,993 |
| 93 | 27 | E N2 | 0,66 | 21 | 0,99 |
| 94 | 26 | E N2 | 0,72 | 59 | 0,996 |
| 95 | 13 | E N1 | 0,88 | 62 | 0,753 |
| 95 | 26 | E N2 | 0,72 | 61 | 0,994 |
| 96 | 14 | E N2 | 0,68 | 193 | 0,993 |
| 97 | 14 | E N2 | 0,68 | 193 | 0,992 |
| 98 | 15 | N1 N2 | 1,13 | 140 | 0,887 |
| 110 | 18 | E N1 | 0,91 | 80 | 0,546 |
| 111 | 18 | E N2 | 0,56 | 22 | 0,939 |
| 111 | 18 | E N1 | 1,17 | 75 | 0,686 |
| 112 | 20 | N1 N2 | 0,48 | 125 | 0,898 |
| 113 | 20 | N1 N2 | 0,57 | 121 | 0,916 |
| 114 | 20 | N1 N2 | 0,45 | 118 | 0,923 |
| 115 | 20 | N1 N2 | 0,51 | 126 | 0,896 |
| 116 | 20 | N1 N2 | 0,42 | 130 | 0,938 |
| 117 | 20 | N1 N2 | 0,5 | 132 | 0,896 |
| 118 | 20 | N1 N2 | 0,62 | 129 | 0,929 |
| 119 | 20 | N1 N2 | 0,57 | 133 | 0,938 |
| 120 | 20 | N1 N2 | 0,6 | 126 | 0,937 |
| 121 | 20 | N1 N2 | 0,44 | 126 | 0,888 |
| 122 | 20 | N1 N2 | 0,54 | 126 | 0,888 |
| 123 | 20 | N1 N2 | 0,5 | 128 | 0,886 |
| 124 | 20 | N1 N2 | 0,61 | 123 | 0,92 |
| 125 | 20 | N1 N2 | 0,35 | 89 | 0,836 |
| 126 | 20 | N1 N2 | 0,5 | 121 | 0,925 |
| 127 | 21 | E N2 | 0,9 | 78 | 0,838 |
| 127 | 26 | E N2 | 0,72 | 60 | 0,995 |
| 128 | 22 | N1 N2 | 0,79 | 29 | 0,864 |
| 128 | 22 | E N2 | 0,31 | 37 | 0,851 |
| 129 | 22 | N1 N2 | 0,8 | 28 | 0,82 |
| 129 | 22 | E N2 | 0,29 | 34 | 0,612 |
| 130 | 22 | E N2 | 0,35 | 27 | 0,963 |
| 131 | 23 | ORF RdRp | 0,68 | 75 | 0,92 |
| 131 | 23 | N1 RdRp | 0,27 | 57 | 0,816 |
| 131 | 23 | E RdRp | 5,04 | 70 | 0,812 |
| 131 | 23 | E ORF | 7,63 | 97 | 0,808 |
| 131 | 23 | E N1 | 19,18 | 65 | 0,55 |
| 131 | 23 | N1 ORF | 0,53 | 75 | 0,515 |
| 132 | 23 | ORF RdRp | 0,68 | 59 | 0,959 |
| 132 | 23 | E RdRp | 3,92 | 58 | 0,949 |
| 132 | 23 | N1 RdRp | 0,26 | 49 | 0,938 |
| 132 | 23 | E ORF | 7,75 | 89 | 0,93 |
| 132 | 23 | E N1 | 12,34 | 77 | 0,612 |
| 132 | 23 | N1 ORF | 0,55 | 74 | 0,595 |
| 133 | 23 | ORF RdRp | 0,57 | 82 | 0,939 |
| 133 | 23 | E ORF | 6,96 | 107 | 0,896 |
| 133 | 23 | E RdRp | 3,95 | 79 | 0,86 |
| 133 | 23 | N1 RdRp | 0,21 | 60 | 0,83 |
| 133 | 23 | N1 ORF | 0,6 | 85 | 0,498 |
| 133 | 23 | E N1 | 10,49 | 80 | 0,472 |
| 134 | 23 | E ORF | 8,39 | 63 | 0,905 |
| 134 | 23 | E RdRp | 4,65 | 46 | 0,859 |
| 134 | 23 | ORF RdRp | 0,65 | 47 | 0,856 |
| 134 | 23 | N1 RdRp | 0,4 | 43 | 0,757 |
| 134 | 23 | E N1 | 11,16 | 58 | 0,568 |
| 134 | 23 | N1 ORF | 0,77 | 61 | 0,538 |
| 135 | 23 | E RdRp | 2,51 | 79 | 0,958 |
| 135 | 23 | ORF RdRp | 0,6 | 85 | 0,957 |
| 135 | 23 | E ORF | 4,52 | 110 | 0,941 |
| 135 | 23 | N1 RdRp | 0,24 | 73 | 0,914 |
| 135 | 23 | N1 ORF | 0,46 | 86 | 0,877 |
| 135 | 23 | E N1 | 7,13 | 94 | 0,791 |
| 136 | 23 | ORF RdRp | 0,59 | 79 | 0,935 |
| 136 | 23 | E ORF | 4,94 | 108 | 0,898 |
| 136 | 23 | N1 RdRp | 0,23 | 66 | 0,881 |
| 136 | 23 | E RdRp | 3,03 | 79 | 0,808 |
| 136 | 23 | N1 ORF | 0,54 | 94 | 0,65 |
| 136 | 23 | E N1 | 8,35 | 93 | 0,571 |
| 137 | 23 | ORF RdRp | 0,56 | 81 | 0,959 |
| 137 | 23 | E ORF | 5,04 | 109 | 0,929 |
| 137 | 23 | E RdRp | 2,64 | 77 | 0,901 |
| 137 | 23 | N1 RdRp | 0,24 | 65 | 0,854 |
| 137 | 23 | E N1 | 7,78 | 91 | 0,711 |
| 137 | 23 | N1 ORF | 0,6 | 86 | 0,68 |
| 138 | 23 | ORF RdRp | 0,61 | 86 | 0,969 |
| 138 | 23 | N1 RdRp | 0,2 | 71 | 0,909 |
| 138 | 23 | E RdRp | 2,63 | 86 | 0,898 |
| 138 | 23 | E ORF | 4,29 | 109 | 0,892 |
| 138 | 23 | E N1 | 8,15 | 94 | 0,772 |
| 138 | 23 | N1 ORF | 0,47 | 92 | 0,748 |
| 139 | 23 | ORF RdRp | 0,66 | 76 | 0,938 |
| 139 | 23 | E RdRp | 3,03 | 75 | 0,896 |
| 139 | 23 | E ORF | 4,94 | 104 | 0,875 |
| 139 | 23 | N1 RdRp | 0,2 | 62 | 0,834 |
| 139 | 23 | N1 ORF | 0,43 | 81 | 0,657 |
| 139 | 23 | E N1 | 10,49 | 83 | 0,657 |
| 140 | 23 | ORF RdRp | 0,69 | 73 | 0,96 |
| 140 | 23 | E ORF | 4,96 | 98 | 0,92 |
| 140 | 23 | E RdRp | 3,39 | 71 | 0,911 |
| 140 | 23 | N1 RdRp | 0,22 | 59 | 0,837 |
| 140 | 23 | E N1 | 11,66 | 81 | 0,587 |
| 140 | 23 | N1 ORF | 0,42 | 80 | 0,574 |
| 141 | 23 | ORF RdRp | 0,55 | 40 | 0,944 |
| 141 | 23 | E ORF | 5,29 | 40 | 0,922 |
| 141 | 23 | E RdRp | 2,94 | 38 | 0,866 |
| 141 | 23 | N1 ORF | 0,38 | 33 | 0,862 |
| 141 | 23 | E N1 | 12,69 | 35 | 0,843 |
| 141 | 23 | N1 RdRp | 0,21 | 32 | 0,714 |
| 142 | 23 | ORF RdRp | 0,71 | 67 | 0,929 |
| 142 | 23 | E ORF | 5,48 | 99 | 0,918 |
| 142 | 23 | E RdRp | 3,64 | 66 | 0,847 |
| 142 | 23 | N1 RdRp | 0,16 | 54 | 0,743 |
| 142 | 23 | E N1 | 16,15 | 71 | 0,446 |
| 142 | 23 | N1 ORF | 0,4 | 78 | 0,333 |
| 151 | 24 | E N2 | 0,68 | 17 | 0,98 |
| 155 | 26 | E N2 | 0,69 | 21 | 0,993 |
| 156 | 26 | E N2 | 0,72 | 19 | 0,996 |
| 157 | 26 | E N2 | 0,74 | 22 | 0,997 |
| 158 | 27 | E N2 | 0,68 | 21 | 0,995 |
| 159 | 27 | E N2 | 0,69 | 20 | 0,997 |
| 160 | 27 | E N2 | 0,68 | 20 | 0,998 |

# R/IIP share, highest F1-score for all WWTP-Lab

Table S2 shows the results of our model for all WWTP-Lab. Displayed are the number of samples, the R/IIP-share of the raw data and the corresponding data quality category (1 = “good” data quality, 2 = “mediocre” data quality, 3 = “bad” data quality). The parameter with the highest F1-score is displayed next to its corresponding F1-score. Delta R/IIP share shows the change in the R/IIP share by excluding all outliers of the parameter with the highest F1-score.

Table S2: Model results of all WWTP and laboratories. NAs for the F1-score and the delta R/IIP share are produced if there is no match between QCP outliers and R/IIP, hence no F1 scores could be calculated.

| **WWTP** | **Laboratory** | **No. Samples** | **Initial R/IIP share** | **Data quality category** | **Highest F1-score parameter** | **Highest F1-score** | **delta R/IIP share** |
| --- | --- | --- | --- | --- | --- | --- | --- |
| 1 | 1 | 21 | 33 | 3 | g_ratios | 0,364 | -0,107 |
| 1 | 6 | 102 | 11 | 1 | PMMOV | 0,143 | -0,063 |
| 1 | 26 | 54 | 19 | 1 | Q | 0,25 | -0,098 |
| 2 | 1 | 24 | 25 | 2 | NA | NA | NA |
| 3 | 1 | 23 | 26 | 2 | EC | 0,286 | -0,125 |
| 4 | 1 | 25 | 24 | 2 | Q | 0,286 | -0,13 |
| 5 | 1 | 21 | 10 | 1 | Q | 0,4 | -0,412 |
| 6 | 1 | 21 | 19 | 1 | Q | 0,4 | -0,211 |
| 7 | 1 | 24 | 29 | 2 | PMMOV | 0,25 | -0,104 |
| 8 | 1 | 25 | 32 | 3 | EC | 0,222 | -0,087 |
| 9 | 1 | 25 | 24 | 2 | NA | NA | NA |
| 10 | 1 | 25 | 20 | 2 | PMMOV | 0,25 | -0,04 |
| 11 | 19 | 90 | 17 | 1 | Q | 0,452 | -0,35 |
| 11 | 26 | 32 | 16 | 1 | NA | NA | NA |
| 16 | 1 | 25 | 24 | 2 | Q | 0,5 | -0,273 |
| 17 | 1 | 23 | 17 | 1 | g_ratios | 0,4 | -0,214 |
| 18 | 1 | 20 | 15 | 1 | NA | NA | NA |
| 19 | 1 | 25 | 28 | 2 | EC | 0,4 | -0,184 |
| 20 | 21 | 98 | 28 | 2 | g_ratios | 0,238 | -0,024 |
| 21 | 1 | 24 | 21 | 2 | g_ratios | 0,333 | -0,164 |
| 22 | 1 | 24 | 17 | 1 | pH | 0,4 | -0,216 |
| 23 | 1 | 16 | 25 | 2 | pH | 0,4 | -0,196 |
| 24 | 1 | 25 | 16 | 1 | NA | NA | NA |
| 25 | 2 | 115 | 39 | 3 | Q | 0,214 | -0,041 |
| 25 | 13 | 129 | 22 | 2 | g_ratios | 0,34 | -0,153 |
| 26 | 2 | 117 | 29 | 2 | Q | 0,195 | -0,061 |
| 26 | 13 | 57 | 26 | 2 | Q | 0,435 | -0,222 |
| 27 | 2 | 109 | 37 | 3 | g_ratios | 0,085 | 0,016 |
| 27 | 13 | 20 | 25 | 2 | PMMOV | 0,571 | -0,329 |
| 29 | 3 | 16 | 31 | 3 | g_ratios | 0,286 | -0,077 |
| 30 | 3 | 19 | 37 | 3 | EC | 0,444 | -0,19 |
| 31 | 3 | 20 | 20 | 2 | g_ratios | 0,571 | -0,406 |
| 34 | 3 | 16 | 12 | 1 | PMMOV | 0,5 | -0,423 |
| 35 | 3 | 20 | 50 | 3 | g_ratios | 0,333 | -0,106 |
| 35 | 11 | 109 | 39 | 3 | g_ratios | 0,241 | -0,028 |
| 36 | 3 | 21 | 38 | 3 | pH | 0,222 | -0,079 |
| 37 | 3 | 16 | 25 | 2 | g_ratios | 0,444 | -0,25 |
| 38 | 3 | 18 | 33 | 3 | NA | NA | NA |
| 40 | 4 | 24 | 33 | 3 | Q | 0,364 | -0,138 |
| 40 | 7 | 20 | 30 | 3 | Q | 0,286 | -0,12 |
| 41 | 4 | 20 | 15 | 1 | NA | NA | NA |
| 41 | 7 | 16 | 50 | 3 | Q | 0,222 | -0,063 |
| 42 | 6 | 68 | 19 | 1 | EC | 0,25 | -0,114 |
| 43 | 5 | 148 | 22 | 2 | EC | 0,326 | -0,156 |
| 44 | 5 | 123 | 20 | 2 | EC | 0,378 | -0,199 |
| 45 | 6 | 169 | 16 | 1 | Q | 0,409 | -0,258 |
| 46 | 6 | 205 | 19 | 1 | pH | 0,255 | -0,11 |
| 47 | 9 | 35 | 29 | 2 | NA | NA | NA |
| 48 | 6 | 28 | 25 | 2 | g_ratios | 0,308 | -0,082 |
| 48 | 8 | 86 | 22 | 2 | g_ratios | 0,296 | -0,129 |
| 49 | 8 | 100 | 12 | 1 | Q | 0,333 | -0,202 |
| 53 | 7 | 15 | 47 | 3 | pH | 0,25 | -0,077 |
| 56 | 7 | 20 | 30 | 3 | CrA | 0,5 | -0,255 |
| 59 | 7 | 17 | 35 | 3 | CrA | 0,4 | -0,143 |
| 61 | 7 | 78 | 21 | 2 | CrA | 0,471 | -0,269 |
| 61 | 8 | 89 | 16 | 1 | pH | 0,273 | -0,136 |
| 62 | 7 | 71 | 32 | 3 | pH | 0,222 | -0,036 |
| 62 | 9 | 71 | 34 | 3 | g_ratios | 0,074 | 0,001 |
| 63 | 7 | 72 | 22 | 2 | CrA | 0,24 | -0,066 |
| 63 | 9 | 78 | 29 | 2 | Q | 0,077 | -0,005 |
| 64 | 7 | 67 | 30 | 3 | g_ratios | 0,438 | -0,206 |
| 64 | 8 | 74 | 23 | 2 | g_ratios | 0,2 | -0,08 |
| 65 | 7 | 75 | 39 | 3 | g_ratios | 0,356 | -0,076 |
| 65 | 26 | 31 | 19 | 1 | PMMOV | 0,286 | -0,138 |
| 66 | 7 | 78 | 33 | 3 | Q | 0,359 | -0,121 |
| 66 | 20 | 84 | 36 | 3 | Q | 0,39 | -0,155 |
| 67 | 21 | 32 | 25 | 2 | g_ratios | 0,222 | 0,107 |
| 68 | 21 | 37 | 19 | 1 | pH | 0,222 | -0,089 |
| 69 | 7 | 20 | 35 | 3 | EC | 0,25 | -0,095 |
| 69 | 21 | 129 | 21 | 2 | g_ratios | 0,267 | -0,052 |
| 70 | 21 | 24 | 29 | 2 | pH | 0,25 | -0,104 |
| 71 | 7 | 41 | 27 | 2 | EC | 0,267 | -0,088 |
| 71 | 22 | 67 | 33 | 3 | PMMOV | 0,083 | -0,002 |
| 72 | 8 | 21 | 19 | 1 | pH | 0,444 | -0,229 |
| 73 | 10 | 95 | 31 | 3 | EC | 0,235 | -0,084 |
| 74 | 8 | 188 | 19 | 1 | PMMOV | 0,286 | -0,147 |
| 75 | 8 | 108 | 20 | 2 | Q | 0,452 | -0,256 |
| 76 | 8 | 23 | 22 | 2 | NA | NA | NA |
| 77 | 8 | 72 | 31 | 3 | pH | 0,083 | 0,008 |
| 77 | 10 | 93 | 26 | 2 | pH | 0,216 | -0,011 |
| 78 | 8 | 71 | 27 | 2 | pH | 0,296 | -0,109 |
| 78 | 10 | 86 | 31 | 3 | Q | 0,205 | -0,008 |
| 79 | 8 | 37 | 38 | 3 | g_ratios | 0,125 | -0,017 |
| 79 | 10 | 59 | 27 | 2 | g_ratios | 0,222 | 0,003 |
| 80 | 8 | 21 | 10 | 1 | Q | 0,5 | -0,444 |
| 80 | 10 | 100 | 28 | 2 | Q | 0,341 | -0,137 |
| 81 | 8 | 23 | 17 | 1 | NA | NA | NA |
| 81 | 10 | 76 | 17 | 1 | g_ratios | 0,417 | -0,279 |
| 82 | 10 | 111 | 33 | 3 | EC | 0,208 | -0,045 |
| 83 | 10 | 91 | 30 | 3 | NA | NA | NA |
| 84 | 10 | 176 | 17 | 1 | EC | 0,4 | -0,238 |
| 85 | 10 | 85 | 35 | 3 | Q | 0,205 | -0,016 |
| 86 | 10 | 155 | 30 | 3 | g_ratios | 0,203 | -0,05 |
| 87 | 9 | 33 | 15 | 1 | g_ratios | 0,286 | -0,147 |
| 89 | 9 | 31 | 13 | 1 | NA | NA | NA |
| 90 | 10 | 193 | 21 | 2 | EC | 0,256 | -0,115 |
| 91 | 9 | 30 | 30 | 3 | NA | NA | NA |
| 92 | 9 | 31 | 16 | 1 | pH | 0,364 | -0,25 |
| 93 | 11 | 117 | 34 | 3 | EC | 0,292 | -0,092 |
| 93 | 26 | 44 | 11 | 1 | Q | 0,2 | -0,095 |
| 93 | 27 | 21 | 14 | 1 | NA | NA | NA |
| 94 | 26 | 59 | 12 | 1 | EC | 0,333 | -0,177 |
| 95 | 13 | 62 | 19 | 1 | NA | NA | NA |
| 95 | 26 | 61 | 16 | 1 | Q | 0,316 | -0,176 |
| 96 | 14 | 194 | 25 | 2 | Q | 0,222 | -0,076 |
| 97 | 14 | 194 | 21 | 2 | Q | 0,25 | -0,086 |
| 98 | 15 | 161 | 22 | 2 | g_ratios | 0,19 | 0,003 |
| 99 | 16 | 208 | 22 | 2 | Q | 0,258 | -0,104 |
| 100 | 16 | 194 | 26 | 2 | Q | 0,19 | -0,059 |
| 101 | 16 | 153 | 14 | 1 | CrA | 0,2 | -0,08 |
| 102 | 16 | 167 | 29 | 2 | Q | 0,14 | -0,031 |
| 103 | 17 | 170 | 14 | 1 | Q | 0,188 | -0,082 |
| 104 | 17 | 170 | 20 | 2 | pH | 0,286 | -0,144 |
| 106 | 17 | 170 | 31 | 3 | PMMOV | 0,161 | -0,039 |
| 107 | 17 | 170 | 21 | 2 | Q | 0,182 | -0,064 |
| 108 | 17 | 171 | 14 | 1 | PMMOV | 0,059 | 0,018 |
| 109 | 17 | 170 | 22 | 2 | pH | 0,163 | 0,039 |
| 110 | 18 | 103 | 24 | 2 | PMMOV | 0,286 | -0,112 |
| 111 | 18 | 111 | 21 | 2 | g_ratios | 0,298 | -0,11 |
| 112 | 20 | 126 | 34 | 3 | g_ratios | 0,204 | -0,072 |
| 113 | 20 | 117 | 29 | 2 | g_ratios | 0,146 | -0,03 |
| 114 | 20 | 119 | 29 | 2 | PMMOV | 0,308 | -0,111 |
| 115 | 20 | 129 | 35 | 3 | g_ratios | 0,203 | -0,027 |
| 116 | 20 | 133 | 35 | 3 | g_ratios | 0,286 | -0,08 |
| 117 | 20 | 131 | 32 | 3 | g_ratios | 0,255 | -0,074 |
| 118 | 20 | 131 | 19 | 1 | g_ratios | 0,162 | -0,031 |
| 119 | 20 | 133 | 32 | 3 | Q | 0,2 | -0,067 |
| 120 | 20 | 127 | 36 | 3 | Q | 0,349 | -0,12 |
| 121 | 20 | 127 | 40 | 3 | g_ratios | 0,161 | -0,012 |
| 122 | 20 | 127 | 35 | 3 | PMMOV | 0,231 | -0,078 |
| 123 | 20 | 129 | 27 | 2 | Q | 0,286 | -0,102 |
| 124 | 20 | 122 | 26 | 2 | pH | 0,341 | -0,155 |
| 125 | 20 | 85 | 29 | 2 | pH | 0,333 | -0,149 |
| 126 | 20 | 122 | 34 | 3 | pH | 0,125 | -0,022 |
| 127 | 21 | 83 | 24 | 2 | g_ratios | 0,216 | 0,025 |
| 127 | 26 | 60 | 23 | 2 | NA | NA | NA |
| 128 | 22 | 72 | 29 | 2 | g_ratios | 0,27 | -0,016 |
| 129 | 22 | 65 | 25 | 2 | Q | 0,182 | -0,034 |
| 130 | 22 | 27 | 22 | 2 | g_ratios | 0,364 | -0,175 |
| 131 | 23 | 72 | 15 | 1 | PMMOV | 0,167 | -0,072 |
| 132 | 23 | 63 | 30 | 3 | g_ratios | 0,133 | 0,088 |
| 133 | 23 | 82 | 15 | 1 | EC | 0,111 | 0,018 |
| 134 | 23 | 46 | 22 | 2 | Q | 0,308 | -0,143 |
| 135 | 23 | 89 | 13 | 1 | PMMOV | 0,4 | -0,342 |
| 136 | 23 | 93 | 17 | 1 | g_ratios | 0,071 | 0,078 |
| 137 | 23 | 88 | 19 | 1 | Q | 0,1 | -0,025 |
| 138 | 23 | 86 | 14 | 1 | g_ratios | 0,231 | -0,102 |
| 139 | 23 | 85 | 7 | 1 | PMMOV | 0,25 | -0,137 |
| 140 | 23 | 74 | 18 | 1 | pH | 0,364 | -0,21 |
| 141 | 23 | 34 | 9 | 1 | Q | 0,4 | -0,29 |
| 142 | 23 | 72 | 22 | 2 | Q | 0,19 | -0,059 |
| 155 | 26 | 21 | 24 | 2 | EC | 0,286 | -0,111 |
| 156 | 26 | 20 | 45 | 3 | g_ratios | 0,364 | -0,131 |
| 157 | 26 | 22 | 18 | 1 | NA | NA | NA |
| 158 | 27 | 21 | 19 | 1 | NA | NA | NA |
| 159 | 27 | 21 | 14 | 1 | NA | NA | NA |
| 160 | 27 | 20 | 15 | 1 | Q | 0,4 | -0,255 |
